# Essential roles of the ANKRD31–REC114 interaction in meiotic recombination and mouse spermatogenesis

**DOI:** 10.1073/pnas.2310951120

**Published:** 2023-11-17

**Authors:** Jiaqi Xu, Tao Li, Soonjoung Kim, Michiel Boekhout, Scott Keeney

**Affiliations:** ^a^Biochemistry, Cell, and Molecular Biology (BCMB) Allied Program, Weill Cornell Graduate School of Medical Sciences, New York, NY 10065; ^b^Molecular Biology Program, Memorial Sloan Kettering Cancer, New York, NY 10065; ^c^HHMI, Memorial Sloan Kettering Cancer Center, New York, NY 10065

**Keywords:** meiosis, homologous recombination, Spo11, Ankrd31, spermatogenesis

## Abstract

Meiotic DNA double-strand breaks (DSBs) initiate homologous recombination and are crucial for ensuring proper chromosome segregation. In mice, ANKRD31 recently emerged as a regulator of DSB timing, number, and location, with a particularly important role in targeting DSBs to the pseudoautosomal regions (PARs) of sex chromosomes. ANKRD31 interacts with multiple proteins, including the conserved and essential DSB-promoting factor REC114, so it was hypothesized to be a modular scaffold that “anchors” other proteins together and to meiotic chromosomes. To determine whether and why the REC114 interaction is important for ANKRD31 function, we generated mice with *Ankrd31* mutations that either reduced (missense mutation) or eliminated (C-terminal truncation) the ANKRD31–REC114 interaction without diminishing contacts with other known partners. A complete lack of the ANKRD31–REC114 interaction mimicked an *Ankrd31* null, with delayed DSB formation and recombination, defects in DSB repair, and altered DSB locations including failure to target DSBs to the PARs. In contrast, when the ANKRD31–REC114 interaction was substantially but not completely disrupted, spermatocytes again showed delayed DSB formation globally, but recombination and repair were hardly affected and DSB locations were similar to control mice. The missense *Ankrd31* allele showed a dosage effect, wherein combining it with the null or C-terminal truncation allele resulted in intermediate phenotypes for DSB formation, recombination, and DSB locations. Our results show that ANKRD31 function is critically dependent on its interaction with REC114 and that defects in ANKRD31 activity correlate with the severity of the disruption of the interaction.

## Significance

Recombination between homologous chromosomes is essential for gamete formation in mammals. Recombination initiates with DNA double-strand breaks (DSBs), whose formation is tightly regulated. The vertebrate-specific ANKRD31 protein is an important part of this regulation in mice and humans and is particularly important for ensuring that the sex chromosomes in males can recombine. ANKRD31 interacts with many different proteins including the REC114 protein, but it has not been established whether these interactions are important for meiosis. We generated mice with targeted mutations in the *Ankrd31* gene that reduce or eliminate the interaction with REC114 without altering interactions with the other known ANKRD31 partners. Analysis of these mice demonstrates that the ANKRD31–REC114 interaction is critical for all ANKRD31 functions in meiosis.

During meiosis, one round of DNA replication is followed by two rounds of chromosome segregation to reduce the chromosome complement. In many species, the segregation of homologous chromosomes during meiosis I requires programmed DNA double-strand breaks (DSBs) to be generated and then repaired by homologous recombination. These DSBs are formed by SPO11 and accessory proteins (1), which in mice include meiosis-specific REC114, MEI4, and IHO1 (orthologs of yeast Rec114, Mei4, and Mer2, respectively) (2–5). Although the major protein players have been known for some time, the physical and functional interactions among them are not well understood, particularly in mammals.

**Fig. 1.**
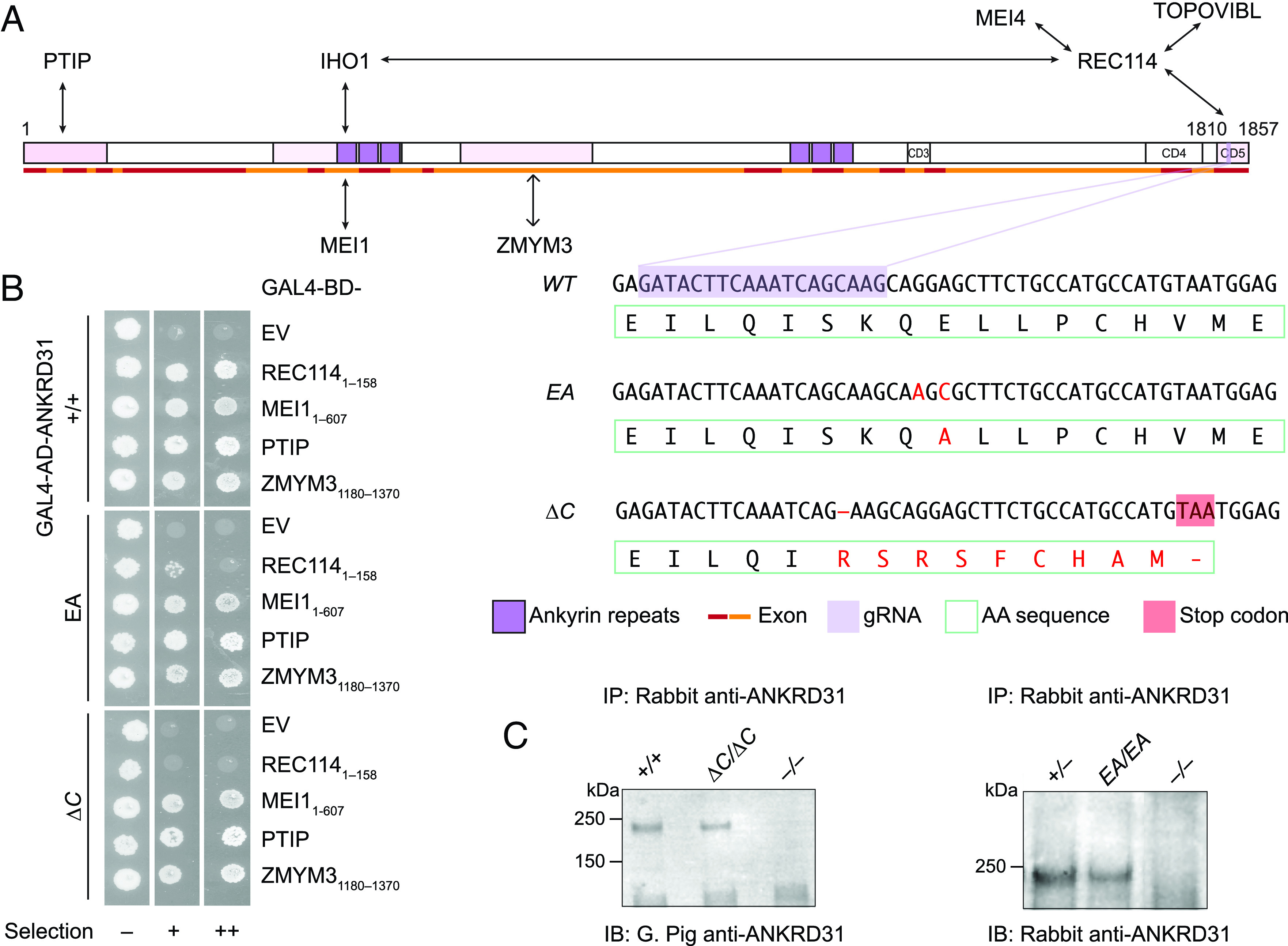
Generation of ANKRD31–REC114 interaction-deficient mice. (*A*) Mouse ANKRD31 domain structure, interactions, and CRISPR/Cas9 guide RNA target. Ankyrin repeat domains (dark purple), conserved domains (CD3–CD5), interacting regions (shaded in pink), exons (red and orange lines), and gRNA sequence (light purple) are indicated. DNA and amino acid sequences of wild-type and mutated alleles are shown below with mutant sequences in red and the premature stop codon in ANKRD31-ΔC shaded in red. (*B*) Y2H interactions of wild-type and mutated ANKRD31 with full-length or parts (indicated) of REC114, MEI1, PTIP, and ZMYM3. Cells express the indicated Gal4 activating domain (AD) and binding domain (BD) fusions. EV, empty vector. “Selection” indicates amino acid dropouts and aureobasidin to detect reporter activation at moderate (+) and high (++) stringency. (*C*) Immunoprecipitation (IP) and immunoblotting (IB) of ANKRD31 from whole-testis extracts of control and mutated mice. Adult mice (>2 mo old) were used in the experiment on the right. Younger mutant mice (1.5 mo old) were used for the experiment on the left to reduce potential effects of the altered testis cellularity in *Ankrd31*^Δ^*^C/^*^Δ^*^C^* adults (*Methods*).

The vertebrate-specific protein ANKRD31 (Ankyrin Repeat Domain Containing 31) was identified as a direct interaction partner of REC114 (6, 7). ANKRD31 contains two ankyrin repeat domains and three additional conserved regions (Fig. 1*A*). The C-terminal-most conserved region (aa 1808–1857) wraps around the N-terminal pleckstrin homology (PH) domain of REC114 as shown by a crystal structure of the complex (6) (*SI Appendix*, Fig. S1*A*). Other segments in ANKRD31 interact with additional partners IHO1, MEI1, PTIP, and ZMYM3 (6–8) (Fig. 1 *A* and *B*).

*Ankrd31*-deficient male mice have delayed DSB formation and defects in DSB repair, accompanied by partially penetrant arrest and apoptosis of spermatocytes during pachynema (6, 7). Because oocytes also show these DSB defects, *Ankrd31^–/–^* females are fertile when young but have reduced oocyte reserve and show premature ovarian failure (6, 7).

*Ankrd31* deficiency also leads to two major changes in DSB locations. First, there is a near complete loss of the high-frequency DSBs in the pseudoautosomal regions (PARs) of the X and Y chromosomes, which are necessary for sex chromosome pairing, recombination, and segregation in spermatocytes (6–8). Consequently, most cells that successfully develop beyond pachynema arrest at metaphase I—probably because their achiasmate sex chromosomes trigger a spindle checkpoint as in other mutants (9, 10)—leading to sterility in males. Second, there is a change in global DSB locations (6, 7), in which the DSB hotspots that are targeted by the PRDM9 histone methyltransferase (11) are joined by additional (“default”) hotspots that occur near promoters and other genomic locations that are typically only targeted for SPO11 activity in the absence of PRDM9 function (12).

During early prophase I, ANKRD31 forms numerous small foci that colocalize with DSB-promoting factors such as REC114, MEI1, and MEI4 on chromosome axes (6–8). These small foci are presumably involved in forming the hundreds of DSBs that are targeted by PRDM9 across the genome. Alterations at these focal sites are also presumably the cause of the changed DSB locations, delayed DSB formation, and defective DSB repair in *Ankrd31* null mutants. In addition, ANKRD31 forms large immunostaining “blobs” on repetitive arrays of the mo-2 minisatellite that are present on the PAR and the centromere-distal ends of chromosomes 4, 9 and 13 (6–8). These blobs also contain REC114, MEI4, MEI1, and IHO1 and are responsible for PAR axis remodeling that enables efficient DSB formation (8). ANKRD31, REC114, and MEI4—but not IHO1—are essential for mo-2 blob formation (6–8).

Given the complexity of ANKRD31 function and the fact that ANKRD31 interacts with multiple meiotic proteins, we hypothesized that ANKRD31 might act as a scaffold that anchors REC114 and other interactors to specific genomic loci at different times, thus regulating DSB formation (6). A key prediction of this hypothesis is that each specific ANKRD31 protein–protein interaction is important for meiosis, but which function(s) of ANKRD31 depends on which interactions is unknown.

To address these issues, we used CRISPR/Cas9 genome editing to alter the last *Ankrd31* exon (encoding the REC114-interaction domain) to generate mutations that disrupt the ANKRD31–REC114 interaction to different extents. Analysis of mice carrying *Ankrd31* mutant alleles in various combinations showed that this interaction is essential for all ANKRD31 functions, with specific functions dependent to different quantitative degrees on the strength of the interaction.

## Result

### Generation of ANKRD31–REC114 Interaction-Deficient Mice.

In the crystal structure for ANKRD31 residues 1808–1857 (ANKRD31_C_) complexed with REC114 residues 1–158 (REC114_N_), the side chain of ANKRD31 Glu-1831 is anchored by two intermolecular salt bridges and one hydrogen bond (6) (*SI Appendix*, Fig. S1*A*). In pulldown assays with recombinant GST-tagged ANKRD31_C_ and purified REC114_N_, substituting alanine for Glu-1831 dramatically reduced the interaction (6). This mutation similarly disrupted the yeast two-hybrid (Y2H) interaction between ANKRD31_C_ and REC114 1–145 without altering the expression of the protein fragments (*SI Appendix*, Fig. S1 *B* and *C*).

We therefore used a guide RNA (Fig. 1*A*) to target Glu-1831 encoded in exon 25 plus a donor DNA containing desired mutations as the repair template to generate a point mutated allele harboring coding sequence mutations G5490A and A5492C yielding a glutamine to alanine substitution at residue 1831 (hereafter *Ankrd31^EA^*). We also fortuitously obtained a truncated allele (*Ankrd31*^Δ^*^C^*) with a 1-bp deletion at position 5,484 that results in a frameshift mutation and premature termination, replacing the last 21 amino acids with 9 extraneous residues (Fig. 1*A*).

We performed Y2H assays to test whether the interaction with REC114_N_ is disrupted by these two mutations when in the context of full-length ANKRD31 protein. EA severely, but not completely, disrupted the interaction, while ANKRD31-ΔC showed no evidence of residual interaction (Fig. 1*B*). The less complete interaction defect for the point mutation when it was in the full-length protein as compared to the short ANKRD31_C_ peptide (6) (*SI Appendix*, Fig. S1*B*) suggests that there may be additional contacts between REC114_N_ and other parts of ANKRD31 (7). If so, the inability of otherwise full-length ANKRD31-ΔC to interact with REC114_N_ indicates that these contacts are not sufficient.

Both ANKRD31-EA and ANKRD31-ΔC proteins were detected at normal levels by immunoprecipitation from whole-testis extracts followed by immunoblotting (Fig. 1*C*), indicating that the mutations did not substantially alter protein stability in vivo. In addition, both mutant proteins maintained Y2H interaction with other known ANKRD31 partners MEI1, PTIP, and ZMYM3 (Fig. 1*B*), which interact with other parts of ANKRD31 (Fig. 1*A*) (8). We conclude that we successfully generated mutations that cause defects to different extents specifically in the ANKRD31–REC114 interaction.

### Hypogonadism and Infertility in an Interaction-Deficient *Ankrd31* Allelic Series.

**Fig. 2.**
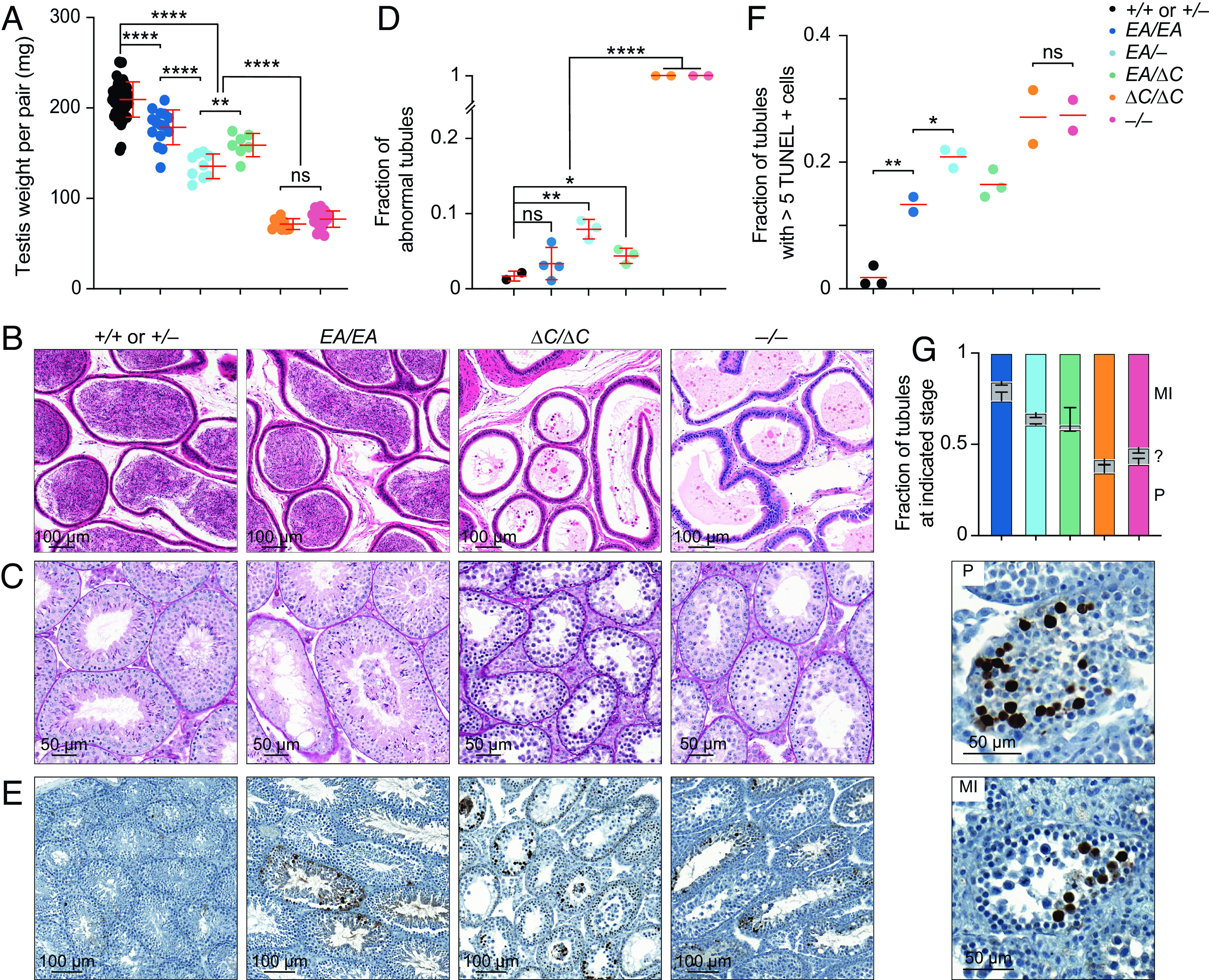
Hypogonadism and infertility in an interaction-deficient *Ankrd31* allelic series. (*A*) Quantification of testis weights (red lines, means ± SD). Mice were 2 to 11 mo old. (*B*) Sections of epididymides from adult mice (2 to 8 mo old), PFA fixed and H&E stained. (*C*) Sections of adult (2 to 8 mo old) seminiferous tubules, Bouin’s fixed and periodic acid Schiff (PAS) stained. (*D*) Quantification of tubules with abnormal spermatogenesis. Each point is the measurement from one animal; red lines are means ± SD. (*E* and *F*) Apoptosis. Adult (2 to 8 mo old) testis sections were stained with TUNEL and hematoxylin. Representative images are shown in panel (*E*), and quantification is presented in panel (*F*), with each point the measurement from one animal; the red lines are means. (*G*) Fraction of apoptotic tubules in mutants according to spermatocyte stage present (mean and SD for at least two experiments). MI, metaphase I; P, pachytene; ?, ambiguous. Representative examples of tubules with pachytene or metaphase I apoptosis are shown at the bottom. The results of two-tailed Mann–Whitney *U* tests in *A*, *D*, and *F* are shown: ns, not significant (*P* > 0.05), ∗*P* ≤ 0.05, ∗∗*P* ≤ 0.01, and ∗∗∗∗*P* ≤ 0.0001. Underlying data for all plots in this and subsequent figures, including exact *P* values, are provided in Dataset S1.

*Ankrd31^–/–^* males are sterile, and their testes are about one-third of the weight of those in control mice (6, 7) (Fig. 2*A*). *Ankrd31*^Δ^*^C/^*^Δ^*^C^* males were also sterile: They had similar testis sizes as *Ankrd31^–/–^* males (Fig. 2*A*), and neither of the two animals tested (9 mo old) sired offspring when bred with fertile females for 8 wk. In contrast, *Ankrd31^EA/EA^* males were fertile, albeit with testis weights significantly lower than wild type (*P* < 0.0001, Student’s *t* test; Fig. 2*A*). Consistent with fertility, epididymal sperm were observed in *Ankrd31^EA/EA^* and control mice but not in *Ankrd31*^Δ^*^C/^*^Δ^*^C^* or *Ankrd31^–/–^* males (Fig. 2*B*).

To explore whether there is a dosage effect of the *Ankrd31^EA^* allele, we also generated compound heterozygotes *Ankrd31^EA/–^* and *Ankrd31^EA/^*^Δ^*^C^*. Heterozygous males of both genotypes had testis weights that were significantly lower than in *Ankrd31^EA/EA^* mice (*P* < 0.0001 and *P* = 0.0244, respectively) but significantly heavier than *Ankrd31*^Δ^*^C/^*^Δ^*^C^* and *Ankrd31^–/–^* males (*P* < 0.0001; Fig. 2*A*). Epididymal sperm were observed in *Ankrd31^EA/–^* and *Ankrd31^EA/^*^Δ^*^C^* males (*SI Appendix*, Fig. S2*A*), and males of both genotypes were able to sire pups, although we cannot rule out that they were subfertile.

Testis sections from control animals had the full array of spermatogenic cells, including spermatocytes and round and elongated spermatids, as expected (Fig. 2*C* and *SI Appendix*, Fig. S2*B*). In contrast, *Ankrd31^–/–^* tubules, as was previously shown (6, 7), contained spermatogonia and primary spermatocytes but were largely if not completely devoid of postmeiotic cells. *Ankrd31*^Δ^*^C/^*^Δ^*^C^* tubules were again similar to *Ankrd31^–/–^* (Fig. 2 *C* and *D* and *SI Appendix*, Fig. S2*B*). In contrast, the majority of *Ankrd31^EA/EA^* tubules appeared normal, and only a small fraction appeared abnormal (emptier and lacking postmeiotic cells). Compound heterozygotes *Ankrd31^EA/–^* and *Ankrd31^EA/^*^Δ^*^C^* both had some normal tubules but also had a larger fraction of abnormal tubules as compared to *Ankrd31^EA/EA^* (Fig. 2*D* and *SI Appendix*, Fig. S2 *B* and *C*).

Meiotic recombination defects cause hypogonadism and sterility because of spermatocyte apoptosis (13). We interpret the less populated tubules in interaction-deficient mutants as those in which apoptosis has already eliminated aberrant cells. Pachytene arrest can be triggered by persistent DSBs or defects in synapsis or meiotic sex chromosome inactivation (14–16). In contrast, metaphase I arrest is typical for mutants that can complete DSB repair but harbor achiasmate chromosomes that trigger a spindle checkpoint (9, 10, 17).

As previously reported, TUNEL staining detected a high frequency (nearly 30%) of apoptotic tubules in *Ankrd31^–/–^* testis sections, displaying partially penetrant pachytene arrest and more fully penetrant metaphase I arrest (6, 7) (Fig. 2 *E* and *F*). *Ankrd31^ΔC/ΔC^* testis sections were comparable (Fig. 2 *E* and *F*). In contrast, *Ankrd31^EA/EA^* testes had a smaller fraction (~10%) of apoptotic tubules, and the compound heterozygotes again showed an intermediate phenotype (~20% apoptotic tubules) (Fig. 2*F* and *SI Appendix*, Fig. S2*D*). In all mutants tested, dying spermatocytes were observed in both pachynema and metaphase I, indicating varying penetrance of arrest at both stages of prophase I (Fig. 2*G*).

### ANKRD31–REC114 Interaction Deficiencies Cause Graded Defects in DSB Formation and Recombination.

To assess meiotic DSB formation and recombination, we immunostained chromosomes for γH2AX, RPA2, DMC1, and RAD51. γH2AX is a phosphorylated form of histone H2AX that is generated by the kinases ATM and ATR during meiotic prophase I in response to SPO11-generated DSBs (18–21). γH2AX initially appears genome-wide during early prophase I and then concentrates in a DSB-independent manner on the silenced X and Y chromosomes (19, 21, 22). RPA2 is a subunit of the single-stranded DNA (ssDNA) binding protein RPA (23). DMC1 (meiosis-specific) and RAD51 (ubiquitously expressed) are strand-exchange proteins homologous to bacterial RecA (23). The ssDNA produced from meiotic DSBs is initially bound by RPA, which is then replaced by RAD51 and DMC1 (24–26). RPA also reaccumulates on recombination intermediates such as displacement (D) loops and/or other DNA structures containing ssDNA. In normal meiosis, chromosome-associated foci of DMC1, RAD51, and RPA appear in leptonema, accumulate to maximal levels in early zygonema, then decline as DSB repair proceeds (27).

**Fig. 3.**
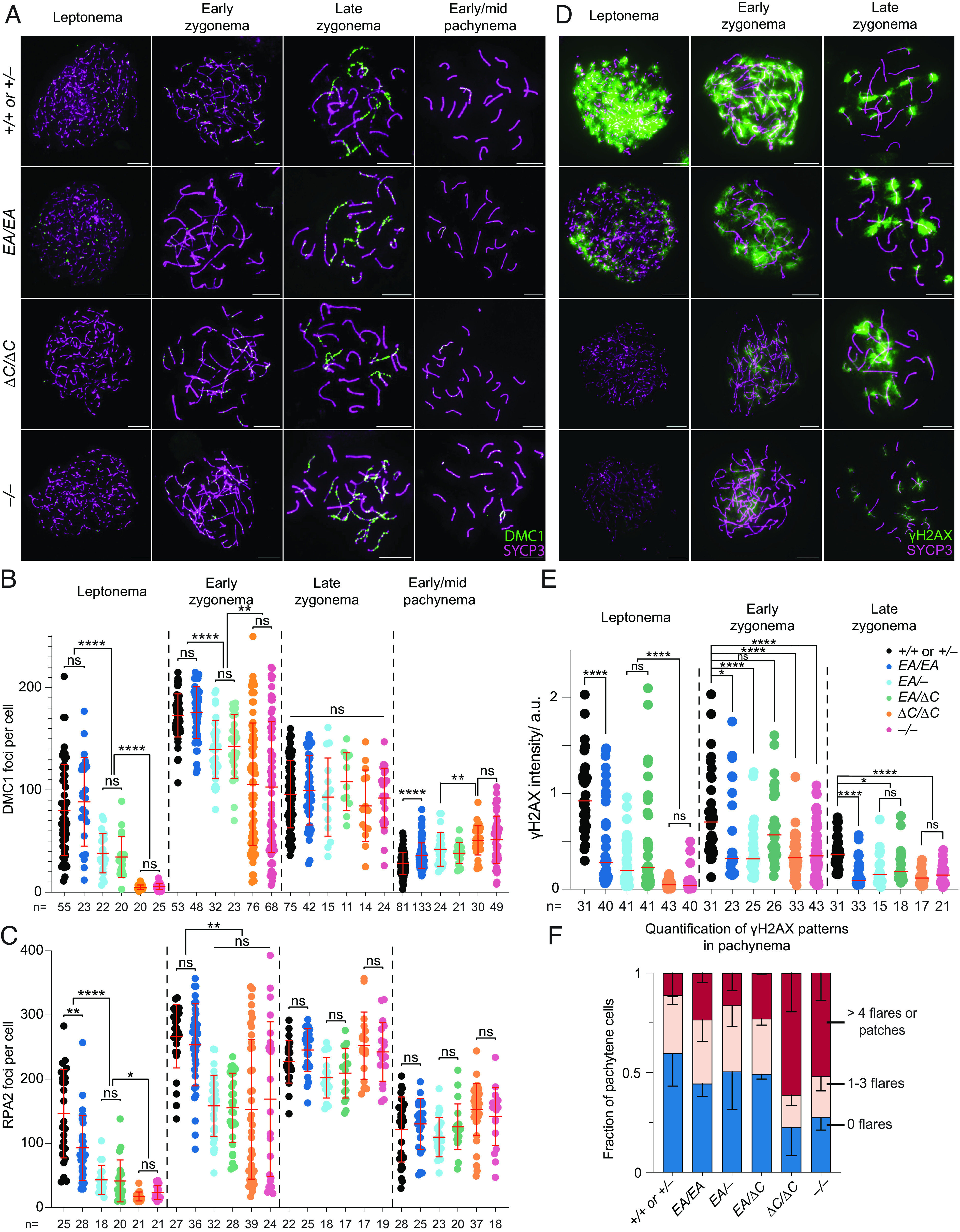
ANKRD31–REC114 interaction deficiencies cause progressive defects in DSB formation and recombination. (*A*) Representative DMC1 staining of spermatocyte chromosome spreads. (Scale bars, 10 μm.) (*B* and *C*) Quantification of focus numbers of DMC1 (*B*) and RPA2 (*C*). Each point is the count from one cell (total cell numbers are given below the graphs) from three or more animals of each genotype. (*D*) Representative γH2AX staining of spermatocyte chromosome spreads. (Scale bars, 10 μm.) (*E*) Quantification of γH2AX staining. The red lines are means. The results of two-tailed Mann–Whitney *U* tests are shown: ns, not significant (*P* > 0.05), ∗*P* ≤ 0.05, ∗∗*P* ≤ 0.01, ∗∗∗*P* ≤ 0.001, and ∗∗∗∗*P* ≤ 0.0001. (*F*) Quantification of γH2AX patterns from at least two mice per genotype. Error bars indicate SD.

As previously shown, recombination focus numbers and γH2AX staining intensity were initially low in *Ankrd31^–/–^* spermatocytes, but they eventually caught up to near normal levels (6, 7) (Fig. 3 *A*–*E* and *SI Appendix*, Figs. S3 and S4 *A* and *B*). Seeing fewer RPA2 foci in early cells suggests that the reduction of RAD51 and DMC1 foci is caused by a delay and/or reduced efficiency of forming cytologically observable sites containing resected DSBs, not simply because of defective loading of the strand-exchange proteins. Thus, it has been interpreted that there is a delay in global DSB formation in the absence of ANKRD31 (6, 7). Additionally, elevated focus numbers and γH2AX staining were observed during pachynema (Fig. 3 *A*–*F* and *SI Appendix*, Figs. S3 and S4 *A*–*C*); these are interpreted as signs of persistent DSBs, suggesting a defect in DSB repair (6, 7).

*Ankrd31^ΔC/ΔC^* spermatocytes behaved comparably to *Ankrd31^–/–^* spermatocytes for all of these DSB markers, at all substages of prophase I (Fig. 3 and *SI Appendix*, Figs. S3 and S4 *B* and *C*). We conclude that the ANKRD31–REC114 interaction is essential for ANKRD31 functions in global DSB formation and recombination.

*Ankrd31^EA/EA^* mice showed a similar but quantitatively milder defect. Compared to control mice, *Ankrd31^EA/EA^* spermatocytes had reduced γH2AX staining throughout early prophase I (Fig. 3 *D* and *E*). RPA2 focus numbers were also lower during leptonema but caught up during later substages, while RAD51 and DMC1 foci remained comparable to controls throughout (Fig. 3 *A*–*C* and *SI Appendix*, Figs. S3 and S4*B*). Again, *Ankrd31^EA/–^* and*Ankrd31^EA/^*^Δ^*^C^* compound heterozygotes showed an intermediate phenotype, with reduced γH2AX staining throughout early prophase I (Fig. 3*E* and *SI Appendix*, Fig. S4*A*) and even fewer DMC1, RAD51, and RPA foci than the *Ankrd31^EA/EA^* homozygous mice (Fig. 3 *B* and *C* and *SI Appendix*, Figs. S3 and S4 *A* and *B*).

In all cases, focus numbers declined as chromosomes synapsed, but to different extents, resulting in more or less similar numbers of foci at late zygonema for all of the genotypes that include the missense allele. However, pachytene cells with apparently normal synapsis also had elevated numbers of DMC1 and RAD51 foci, with the degree of elevation correlating with the severity of the REC114 interaction defect (Fig. 3*B* and *SI Appendix*, Fig. S4*B*). We conclude that attenuating the ANKRD31–REC114 interaction causes a delay in global DSB formation and a defect in completion of recombination that are both quantitatively more modest than when the interaction is more completely disrupted.

We additionally examined crossing-over on autosomes by staining for MLH1, a component of the Holliday junction resolvase (28, 29). Pachytene cells with complete autosome synapsis had similar numbers of MLH1 foci in all genotypes (means of 24.6 in wild type, 24.3 in *Ankrd31^EA/EA^*, 25.1 in *Ankrd31^EA/–^*, 24.3 in *Ankrd31^EA/^*^Δ^*^C^*, 24.4 in *Ankrd31*^Δ^*^C/^*^Δ^*^C^*, and 24.8 in *Ankrd31^–/–^*; *SI Appendix*, Fig. S5 *A* and *B*). This analysis did not reproduce the small but statistically significant increase (6) or decrease (7) in MLH1 foci previously reported for *Ankrd31^–/–^*, but similar to previous results, a small increase in the number of cells with at least one autosome lacking an MLH1 focus was seen in all mutants (*SI Appendix*, Fig. S5*C*; *P* = 0.034 for *Ankrd31^ΔC/ΔC^* and 0.042 for *Ankrd31^–/–^*, Fisher’s exact test).

### Sex Chromosome Pairing Relies on ANKRD31–REC114 Interactions.

**Fig. 4.**
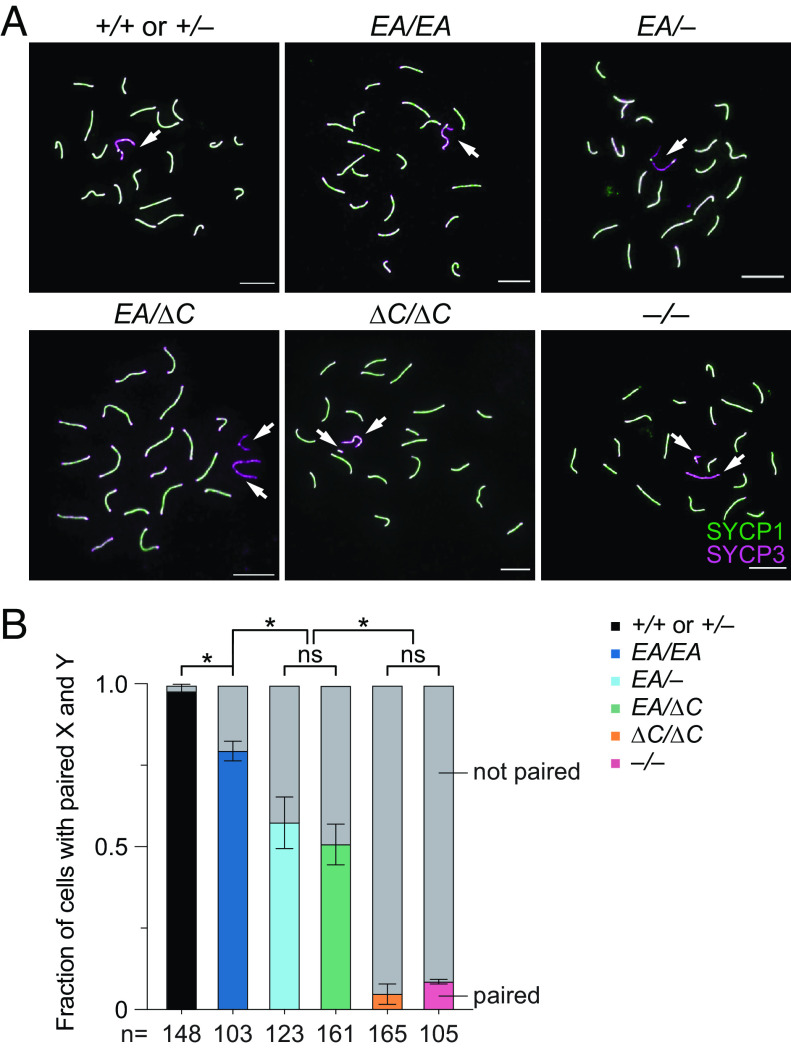
Sex chromosome pairing relies on ANKRD31–REC114 interactions. (*A*) Representative pachytene cell chromosome spreads stained with anti-SYCP3 and anti-SYCP1 antibodies. (Scale bars, 10 μm.) Arrows indicate sex chromosomes. (*B*) Quantification of X-Y pairing frequency at pachynema, based on immunofluorescence for SYCP3 and either SYCP1 or γH2AX, for three animals of each genotype. Total cell numbers are indicated under the graph. The results of Student’s *t* tests after arc sine transformation are shown: ns, not significant (*P* > 0.05), ∗*P* ≤ 0.05.

*Ankrd31^–/–^* metaphase I cells frequently had unaligned chromosomes, and sex chromosomes failed to pair in 92% of otherwise normal-looking pachytene cells (6, 7) (Fig. 4). This high frequency of unpaired and/or achiasmate sex chromosomes is the likely cause of the highly penetrant metaphase I arrest in *Ankrd31^–/–^* mutants.

In keeping with the similarly high frequency of metaphase I arrest as *Ankrd31^–/–^* (Fig. 2*G*), *Ankrd31^ΔC/ΔC^* spermatocytes showed 95.2% of sex chromosomes remaining unpaired in pachynema (Fig. 4). These defects appear to reflect reduced pairing efficiency in the mutants rather than slower pairing that is still efficient because if sex chromosome pairing were simply slower in *Ankrd31^ΔC/ΔC^* and *Ankrd31^–/–^*, we would expect a greater fraction of pachytene cells with paired XY to be in late pachynema than early/mid pachynema. However, in *Ankrd31^ΔC/ΔC^* and *Ankrd31^–/–^* mice, 100% (4 out of 4 cells examined) and 89% (8 out of 9) pachytene cells with paired XY were in early/mid pachynema, respectively. In comparison, in wild type or heterozygous controls, 54% (32 out of 59) and 46% (27 out of 59) of pachytene cells with paired X and Y were in early/pachynema and late pachynema, respectively.

*Ankrd31^EA/EA^* had a much more modest defect, with only 20.6% of sex chromosomes unpaired, while *Ankrd31^EA/–^* and *Ankrd31^EA/^*^Δ^*^C^* compound heterozygotes again had an intermediate defect, with about 50% of sex chromosomes unpaired (Fig. 4). Therefore, the likelihood of successful sex chromosome pairing also correlates with the strength of the ANKRD31–REC114 interaction.

### Altered REC114 and ANKRD31 Localization in Interaction-Deficient Mice.

**Fig. 5.**
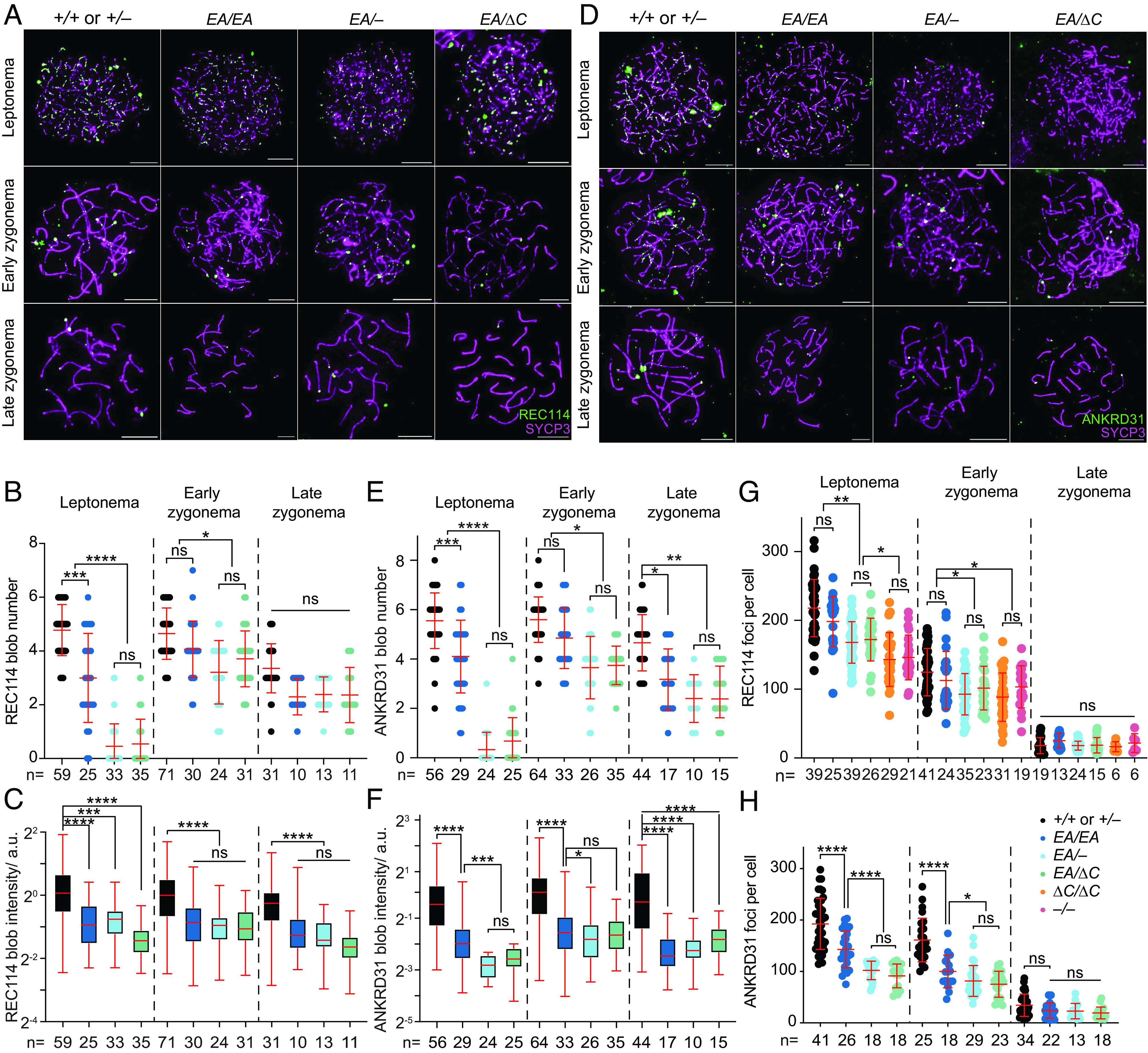
Altered REC114 and ANKRD31 localization in interaction-deficient mice. (*A* and *D*) Representative images of REC114 (*A*) or ANKRD31 (*D*) staining of early prophase spermatocyte chromosome spreads. (Scale bars, 10 μm.) (*B*, *C*, *E*, *F*, *G*, and *H*) Quantification of the REC114 blob number (*B*) and intensity (*C*); ANKRD31 blob number (*E*) and intensity (*F*); and numbers of small foci of REC114 (*G*) or ANKRD31 (*H*) from two or more animals of each genotype. The total cell numbers analyzed are given below each plot. Each point in the scatterplots is the measurement for one cell; red lines indicate means ± SD. In the boxplots (*C* and *F*), the intensities of blobs are normalized to the median blob intensity in the wild type in early zygonema. The boxes indicate the median, 25th, and 75th percentiles, and the whiskers indicate the 10th and 90th percentiles. The results of negative binomial regression tests (*B* and *E*) or two-tailed Mann–Whitney *U* tests (*C*, *F*, *G*, and *H*) are shown: ns, not significant (*P* > 0.05), ∗*P* ≤ 0.05, ∗∗*P* ≤ 0.01, ∗∗∗*P* ≤ 0.001, and ∗∗∗∗*P* ≤ 0.0001.

ANKRD31 is required for the formation of immunostaining blobs of REC114 and other proteins on mo-2 minisatellite arrays on the PAR and elsewhere (Introduction). We therefore asked whether the ANKRD31–REC114 interaction itself is specifically required. When chromosome spreads were stained for REC114, blobs were reduced in number and intensity in all of the *Ankrd31* mutants, with the severity of the defect correlating with the strength of interaction defect (Fig. 5 *A*–*C*). ANKRD31 blobs showed similar decreases (Fig. 5 *D*–*F*).

REC114 and ANKRD31 also extensively colocalize with one another in numerous small foci spread across the chromatin (6, 7). The small REC114 foci still form in the absence of *Ankrd31* but are reduced in number and intensity during leptonema and early zygonema (6, 7) (Fig. 5 *A* and *G*). Not surprisingly then, the smaller REC114 foci were still present in our nonnull *Ankrd31* mutants. Fewer REC114 foci were observed in the truncation mutant at leptonema and early zygonema, similar to the pattern previously shown for *Ankrd31^–/–^* spermatocytes. However, there was no obvious decrease in *Ankrd31^EA/EA^* cells, and the compound heterozygotes again showed an intermediate phenotype, with a modest decrease in focus numbers (Fig. 5 *A* and *G*).

The small foci of ANKRD31 were more strongly affected by the *Ankrd31* mutations. *Ankrd31^EA/EA^* cells had significantly fewer ANKRD31 foci at leptonema and early zygonema, and the compound heterozygotes were still further decreased (Fig. 5 *D* and *H*). Thus, even though the interaction-defective mutant proteins accumulate to normal levels in testes, their localization on chromatin is substantially altered. These findings confirm and extend prior observations and indicate that the ANKRD31–REC114 interaction is important for assembly both of the large immunostaining structures on mo-2 regions and of smaller foci genome-wide.

### The DSB Landscape Is Shaped by the ANKRD31–REC114 Interaction.

In most mammals, DSB locations are controlled by PRDM9, which binds to specific DNA sequences and methylates nearby nucleosomes on histone H3 lysines 4 and 36, thereby targeting SPO11 (11). By single-stranded DNA sequencing (SSDS) of DMC1-bound DNA, most DSB hotspots genome-wide overlap with sites of PRDM9-dependent H3K4me3 (12, 30). In the absence of *Prdm9*, DSBs are instead directed to other H3K4me3-modified loci (default hotspots) such as transcription promoters and CpG islands (30).

ANKRD31 is important for establishing normal DSB distributions (6, 7). *Ankrd31^–/–^* spermatocytes have little or no SSDS signal in the PAR or PAR-proximal hotspots and there is a mixed usage of both PRDM9-targeted and default hotspots elsewhere in the genome (6, 7). Therefore, we asked whether disruption of the ANKRD31–REC114 interaction would alter meiotic DSB locations.

To do this, we performed Exo7/T-seq [modified from S1-seq (31, 32) and END-seq (33)] to map DSBs genome-wide from testes of 14.5 dpp juvenile mice. In this method, high-molecular-weight genomic DNA embedded in agarose is digested with *E*scherichia* coli* exonuclease VII and exonuclease T to remove the single-stranded DNA from resected DSBs; then, sequencing adapters are ligated to the blunted DNA ends (*SI Appendix*, Fig. S6*A*). Two biological replicates were generated for each genotype and averaged (*SI Appendix*, Fig. S6*B*).

**Fig. 6.**
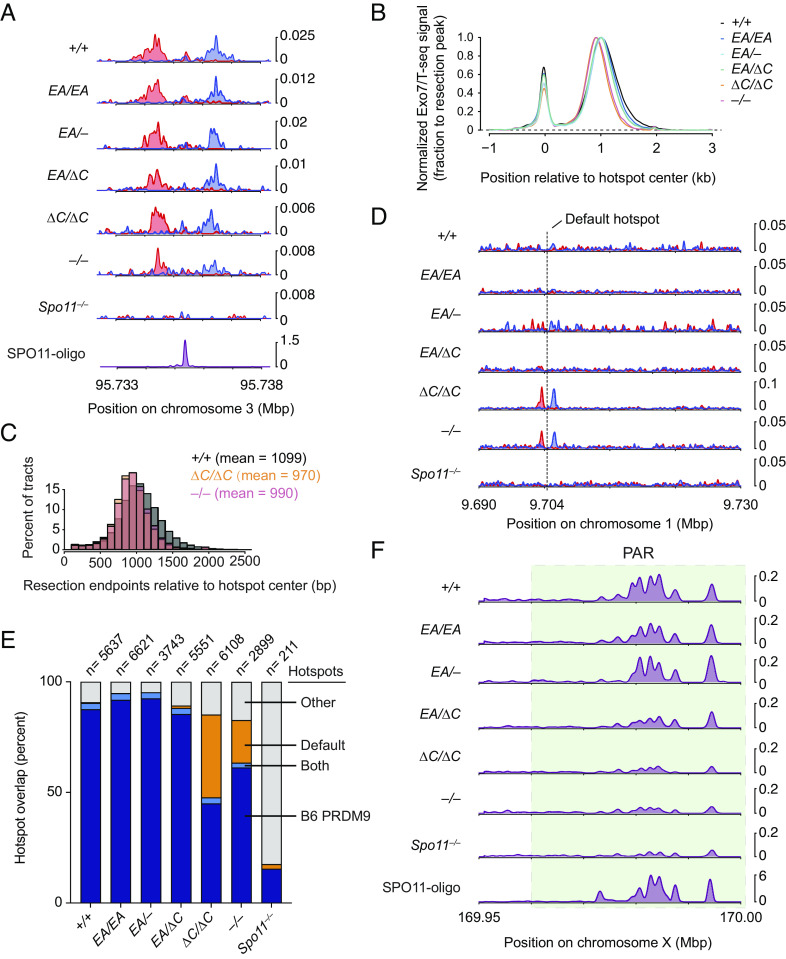
The DSB landscape is shaped by the ANKRD31–REC114 interaction. (*A*) Strand-specific Exo7/T-seq [reads per million mapped reads (RPM)] at a representative DSB hotspot. Top-strand reads are shown in blue, bottom-strand reads in red. Data are smoothed with a 151-bp Hanning window. The SPO11-oligo profile is shown below (34). (*B*) Averaged Exo7/T-seq signals around PRDM9-directed hotspots [n = 13,960 SPO11-oligo hotspots in the C57BL/6J strain (34)]. The bottom-strand reads were flipped and combined with the top-strand reads and normalized to the peak height of resection endpoints (*Methods* and *SI Appendix F*ig. S6*D*). (*C*) Comparison of resection length distributions. (*D*) Strand-specific Exo7/T-seq around a representative default hotspot. Data in 10 bp bins are smoothed with a 51-bin Hanning window. (*E*) Overlap of Exo7/T-seq peak calls in the indicated genotype with either PRDM9-directed SPO11-oligo hotspots (34) or the top 10,000 hottest default hotspots called using SSDS in *Prdm9^–/–^* mice (12). Underlying data for peak calls and overlaps are provided in *SI Appendix T*able S1. (*F*) Exo7/T-seq at the PAR (green shaded region). Sequencing reads from top and bottom strands were combined. Data in 40 bp bins are smoothed with a 51-bin Hanning window.

In wild-type mice, Exo7/T-seq yielded the expected enrichment of top-strand reads to the right of PRDM9-specified hotspots and bottom-strand reads to the left (reflecting 5′→3′ resection rightward and leftward, respectively) along with a central signal derived from recombination intermediates (*SI Appendix*, Fig. S6*A*) (32, 33). These resection and central signals were retained in *Ankrd31^–/–^*, consistent with previous SSDS experiments (6, 7), as well as in *Ankrd31*^Δ^*^C/^*^Δ^*^C^* and the other *Ankrd31* mutant genotypes tested (Fig. 6 *A* and *B*). Interestingly, a modest (~100 nucleotide) decrease in average resection lengths was observed in *Ankrd31*^Δ^*^C/^*^Δ^*^C^* and *Ankrd31^–/–^* (Fig. 6 *B* and *C* and *SI Appendix*, Fig. S6 *C* and *D*).

As previously shown using SSDS (6, 7), *Ankrd31^–/–^* mutants displayed pronounced DSB signals at default hotspots in addition to PRDM9-directed hotspots (Fig. 6*D*). *Ankrd31*^Δ^*^C/^*^Δ^*^C^* likewise showed elevated use of default hotspots (Fig. 6*D*), consistent with its close similarity to *Ankrd31^–/–^* for other phenotypes.

To explore default hotspot usage more closely, we called peaks from Exo7/T-seq signals from each genotype indicated and counted their overlap with either PRDM9-targeted hotspots [n = 13,960 from SPO11 oligo sequencing (34)] or the 10,000 hottest of previously defined default hotspots (12) (Fig. 6*E* and *SI Appendix*, Table S1). As a control for meiotic DSB-independent sequencing background, we included *Spo11^–/–^* mutants. *Ankrd31^–/–^* and *Ankrd31*^Δ^*^C/^*^Δ^*^C^* mutants showed an obvious elevation of default hotspot usage compared to other genotypes tested (Fig. 6*E*). The *Ankrd31^EA/EA^*, *Ankrd31^EA/–^*, and *Ankrd31^EA/^*^Δ^*^C^* mutants behaved broadly similarly to wild type.

*Ankrd31^–/–^* mutants displayed little if any of the high-level Exo7/T-seq signals normally found in the PAR and were similar to the *Spo11^–/–^* control (Fig. 6*F*), consistent with prior SSDS studies (6, 7). *Ankrd31*^Δ^*^C/^*^Δ^*^C^* mutants were similar (Fig. 6*F*). This finding is consistent with the results that sex chromosomes rarely pair in these two mutants. In contrast, *Ankrd31^EA/EA^* and the two compound heterozygotes displayed strong Exo7/T-seq signals in the PAR regions, broadly similar to control mice.

## Discussion

Although multiple axis-associated proteins are now known to promote or control meiotic DSB formation, recombination, and repair in mice, it remains poorly understood how the interactions among these proteins contribute to their functions. Unlike autosomes, which are homologous along their entire lengths, sex chromosomes in males of most mammals only share a small homologous region that, at around 700 kb, is exceptionally small in mice (35, 36). To ensure sex chromosome pairing, the PAR must form DSBs at a much higher frequency per kb than on autosomes. To achieve this, there is rearrangement of higher-order structure of the PAR chromatin, concomitant with highly enriched pro-DSB proteins (blobs) including ANKRD31 and REC114 (8).

ANKRD31 emerged recently as playing a unique set of roles: On the PAR, it is essential for high-level DSB formation, while globally, it promotes normal timing and locations of DSBs but is dispensable for DSB formation per se. Because ANKRD31 interacts with multiple proteins that function in meiosis, including REC114, IHO1, MEI1, ZMYM3, and PTIP, it has been argued that it acts as a modular scaffold that anchors other proteins together and to meiotic chromosomes (6–8). Our findings are consistent with this interpretation and show that specifically disrupting the ANKRD31–REC114 interaction to different extents proportionately affected ANKRD31 function in vivo. *Ankrd31*^Δ^*^C/^*^Δ^*^C^* males showed phenotypes that were comparable to *Ankrd31^–/–^* in every aspect that we characterized. It is striking that losing just the C-terminal 1.65% of the protein, with all of the tested interactions between ANKRD31 and its partners other than REC114 remaining intact, leads to a complete loss of function. This finding emphasizes the importance of the ANKRD31–REC114 interaction. Defects in *Ankrd31^EA/EA^* mutants were comparatively much milder, even though the ANKRD31–REC114 interaction was severely disrupted as measured by Y2H assays. We speculate that multivalent interactions involving networks of ANKRD31-interacting proteins may partially compensate in vivo for attenuation (but not elimination) of the specific interaction with REC114.

ANKRD31 and REC114 are interdependent for their full level of chromatin association in both small foci and large blobs (6–8), and our results show that interactions of ANKRD31 with its other partners are not sufficient in the absence of REC114 interaction. This suggests that ANKRD31 and REC114 and their interaction are central to assembly of blobs on PAR and other mo-2 regions. In *Saccharomyces cerevisiae,* heterotrimeric Rec114–Mei4 complexes or homotetramers of Mer2 (homologous to IHO1 in mice) bind DNA in a highly cooperative manner in vitro, forming large nucleoprotein condensates containing many protein copies (37). A minimal trimerization domain from both yeast Rec114–Mei4 and mouse REC114–MEI4 is sufficient for condensate formation (37), suggesting a possible connection between the condensates characterized for the yeast proteins and the formation of small foci and blobs in vivo in mice. Although a detailed understanding of the mechanism of mo-2-associated blob formation remains elusive, we consider it likely that the multivalent ANKRD31 scaffold works interdependently with its binding partners to assemble condensates on mo-2 chromatin.

There is an interesting apparent distinction between PAR DSB patterns (which appear broadly normal in *Ankrd31^EA/EA^*; Fig. 6*F*) and sex chromosome pairing (which is substantially impaired; Fig. 4*B*). However, the sequencing assay is not calibrated relative to a standard, so it cannot be used to estimate absolute DSB numbers, and our data therefore cannot definitively establish that there is no effect of the EA mutation on DSB formation. Moreover, there was a clear decrease in ANKRD31 and REC114 blob intensity in this mutant (i.e., the amount of protein accumulating on mo-2-containing regions such as the PAR) (Fig. 5 *C* and *F*). Therefore, it is possible that there is a partial defect in PAR DSB formation (reduced frequency, delayed timing, or both) in *Ankrd31^EA/EA^*. Alternatively, however, it might be that attenuating the ANKRD31-REC114 interaction reduces the recombination efficiency for those DSBs that are successfully made in the PAR and/or alters the ability of recombining PARs to stabilize pairing via synapsis.

Since the function of ANKRD31 in the PAR is important in male but not female meiosis, ANKRD31 deficiency causes sexually dimorphic phenotypes: Spermatogenesis fails catastrophically, but oogenesis proceeds, albeit greatly diminished. In clinical studies, two heterozygous variants [p.Gln 329* and c.1565-2A>G (splice donor before exon 11)] in *ANKRD31* were found in patients with premature ovarian insufficiency (POI) (38). Both variants are predicted to result in early protein truncation that should disrupt ANKRD31 interactions with most or all of its known partners, including REC114. The POI resembles known features of the mouse *Ankrd31* null and truncation mutants, suggesting that ANKRD31 functions are conserved between mouse and human. Since these *ANKRD31* mutations cause POI when heterozygous, the truncated proteins may have dominant interfering effects on human oogenesis, or *ANKRD31* function may be dosage sensitive—analogous to but more pronounced than what we observed in mouse spermatogenesis.

Another woman has been reported to be homozygous for a C to T splicing donor mutation in *ANKRD31* (39). This mutation is predicted to cause a C-terminal truncation lacking exons 24 and 25 (106 amino acids), which is larger than our mouse *Ankrd31*^Δ^*^C^* truncation. No published clinical information is available for this individual, but our findings, combined with those of Wang et al. (38), raise the possibility that she may be predisposed to POI. These clinical findings highlight the importance of understanding the molecular functions of ANKRD31 in meiosis.

## Methods

### Mice.

Mouse experiments were approved by the Memorial Sloan Kettering Cancer Center (MSK) Institutional Animal Care and Use Committee (IACUC) and followed US federal regulatory standards. The previously described *Ankrd31^–^* allele (*Ankrd31^em1Sky^,* MGI: 6343226) carries insertion of an A residue in exon 3, causing frameshift and premature termination (6). Interaction-deficient mice were generated by the MSK Mouse Genetics core facility by targeting exon 25 (*SI Appendix*). Mice were genotyped using PCR on cut toes lysed in Direct Tail lysis buffer (Viagen) with primers as indicated in *SI Appendix*, Table S2.

### Immunoprecipitation and Immunoblot Analysis of ANKRD31.

Because of the reduced size and altered cellularity of testes from adult *Ankrd31*^Δ^*^C/^*^Δ^*^C^* mice, we used testes of younger mutant mice (1.5 mo old) to assess protein levels (Fig. 1 *C*, *Left*). We used adult testes for ANKRD31-EA (Fig. 1 *C*, *Right*) because the missense mutation causes less of a reduction in testis weight. The slight decrease in ANKRD31-EA levels compared to the littermate control may be a consequence of the smaller testes in the mutant. Given that the mutants have much smaller testes and fewer spermatocytes (more apoptotic spermatocytes) as compared to wild type, our observation of comparable or only slightly reduced protein expression supported our conclusion that the mutations did not grossly destabilize ANKRD31 protein. Testis extracts were treated with Benzonase to degrade DNA and immunoprecipitated with [guinea pig anti-ANKRD31, rabbit anti-ANKRD31, or rabbit anti-REC114 (6)] and Protein A Dynabeads. Eluted proteins were separated by SDS-PAGE, transferred to polyvinylidene difluoride (PVDF) membranes, and detected with guinea pig or rabbit anti-ANKRD31 primary antibodies and horseradish peroxidase (HRP) conjugated secondary antibodies (details in *SI Appendix, Methods*).

### Immunostaining of Spermatocyte Chromosome Spreads.

Chromosome spreads were prepared by hypotonic lysis in the presence of paraformaldehyde from cell suspensions generated by enzymatic digestion of decapsulated testes with collagenase and trypsin (details in *SI Appendix, Methods*). Slides were blocked for 30 min at room temperature in a Coplin jar with 100 mL fresh block solution [1× PBS with 0.05% Tween-20 and 3 mg/mL bovine serum albumin (BSA)]. Slides were incubated with 80 µL primary antibody in block solution and covered by parafilm overnight at 4 °C in a humid chamber. Slides were washed 3 × 5 min in block solution, then incubated with 80 µL secondary antibody in block solution for 30 min in a humid chamber at room temperature. Slides were then washed 3 × 5 min in the dark with block solution, rinsed in milliQ H_2_O, and mounted with Vectashield containing DAPI. Primary and secondary antibodies are listed in *SI Appendix*, Table S3. Images were acquired on a Zeiss Axio Observer Z1 Marianas Workstation, equipped with an ORCA-Flash 4.0 camera, illuminated by an X-Cite 120 PC-Q light source, with 100 × 1.4 NA oil immersion objective. Marianas Slidebook (Intelligent Imaging Innovations, Denver Colorado) software was used for acquisition.

To quantify foci of ANKRD31, REC114, DMC1, RAD51, RPA, and MLH1, single cells were manually cropped and counted in Fiji. For ANKRD31 and REC114 blobs, overlap with SYCP3 and fluorescence intensity were determined with a thresholding algorithm regardless of meiotic stage, and a difference of Gaussian (DoG) blur was used to isolate REC114 or ANKRD31 foci. Scripts are available on Github: https://github.com/Boekhout/ImageJScripts.

For γH2AX intensity analysis, cells were substaged manually by SYCP3 staining. Regions of interest were drawn, and integrated density was measured. A region containing no cells was measured for background subtraction. To combine measurements from different experiments, the integrated density was normalized to the mean integrated intensity of wild-type leptotene cells within each experiment.

### Exo7/T-seq.

Exo7/T-seq combines elements of previously described S1-seq (31, 32) and END-seq (33) methods. Specifically, it uses *E. coli* exonuclease VII and exonuclease T to remove ssDNA, like END-seq, but uses the methods for genomic DNA preparation, adapter ligation, and sequencing library preparation from S1-seq. Full details are provided in *SI Appendix*. Briefly, high-molecular-weight genomic DNA was extracted from enzymatically dissociated testicular cells embedded in agarose plugs and then digested with exonuclease VII and exonuclease T. Biotinylated adapters were ligated to the blunted ends; then, the DNA was extracted from the plugs and sheared by sonication, and ligated fragments were purified using streptavidin beads. A second adapter was ligated to the sheared end of each fragment, and the resulting DNA library was amplified by low-cycle-number PCR and then sequenced on the Illumina HiSeq platform in the Integrated Genomics Operation at MSK. We obtained paired-end reads of 50 bp.

Bioinformatic analysis was performed as described previously (31, 32). Briefly, adapter sequences were trimmed and reads were mapped to mouse reference genome mm10 using Bowtie2 (40). Exo7/T-seq signal from the top and bottom strands was averaged, co-oriented, and plotted for each genotype. The averaged profile was smoothed with a 151-bp Hanning filter. Plotting the representative Exo7/T-seq signal from each genomic location was done with the indicated smoothing window and bin sizes. Differences in background levels between samples, likely because of variation in testis cellularity, cause genotype-independent variation in absolute signal level (32) (*SI Appendix*, Fig. S6*D*). Therefore, to compare spatial patterns of resection in Fig. 6*B*, the average profile for each genotype was normalized to the peak height of resection endpoints. To do this, an estimated background was removed by subtracting the value 2,500 bp away from the hotspot center. The background-subtracted profile was then normalized to the maximum value between 100 and 2,500 bp. Negative values were set as zero for plotting purposes. To plot histograms of resection lengths, an estimated background value was removed by subtracting the signal 2.5 kb away from the hotspot center. By setting values for positions 100 bp and >2.5 kb to zero, the signal near and farther from the hotspot core was eliminated. Fractions of remaining total signal were calculated every 100 bp and plotted. Methods details are provided in *SI Appendix*.

## Supplementary Material

Appendix 01 (PDF)

Dataset S01 (XLSX)

## Data Availability

Exo7/T-seq sequencing data have been deposited in the Gene Expression Omnibus (GEO) repository under accession number GSE229450 (41). Underlying data for all plots, including exact *P* values, are provided in Dataset S1.
