# Supplementary material for: Essential roles of the ANKRD31–REC114 interaction in meiotic recombination and mouse spermatogenesis
Source: Proc Natl Acad Sci U S A. 2023 Nov 17;120(47):e2310951120. doi: 10.1073/pnas.2310951120 (PMC10666023; doi:10.1073/pnas.2310951120)
Supplement: Supplementary file 1 — Appendix 01 (PDF) [file pnas.2310951120.sapp.pdf]

Supplemental information for:

Essential roles of the ANKRD31–REC114 interaction in meiotic recombination and mouse spermatogenesis

Jiaqi Xu, Tao Li, Soonjoung Kim, Michiel Boekhout, and Scott Keeney

This pdf contains:

Supplemental Methods

Supplemental References

Supplemental Figures S1-S6

Supplemental Tables S1-S3

Additional information, in a separate .xls file:

Supplemental File 1

Supplemental Methods

Mice

Animals were fed regular rodent chow with ad libitum access to food and water. To generate targeted Ankrd31 mutations, a guide RNA cassette with sequence (5′-GATACTTCAAATCAGCAAGC) and single-stranded donor DNA (5′-GAGTCGATGCAAACCATCCCTCATTATCTACAGATAAAAGAGATACTTCAAATCAGCAAGCAGGAGCTTCTGCCATGCCATGTAATGGAGCAGCATTGGAAGTTCTATGTGGGACGCTC) harboring desired missense mutations were microinjected using conventional techniques (41) into pronuclei of CBA/J × C57BL/6J F2 hybrid zygotes generated by crossing CBAB6F1/J hybrid females with C57BL/6J males. Founder mice (CBA/J × C57BL/6J F2 hybrid) were crossed to C57BL/J6 mice purchased from Jackson Laboratories to obtain germline transmission, then heterozygous animals were backcrossed to C57BL/J6 for at least 3 generations. The systematic names are Ankrd31em3Sky for the EA allele (MGI: 7428356) and Ankrd31em4Sky for the ΔC allele (MGI:7428358). *Ankrd31EA/EA* animals were generated by crossing *Ankrd31+/EA* heterozygous mice with *Ankrd31+/EA* heterozygous or *Ankrd31EA/EA* mice. *Ankrd31*ΔC/ΔC animals were generated by crossing *Ankrd31+/*ΔC heterozygous mice. *Ankrd31–/–* homozygous animals were generated as described (6).

Yeast two-hybrid assays

Genes were amplified by PCR from a testis cDNA library and digested by EcoR I (NEB) and Nde I (NEB), and then cloned into either pGADT7 or pGBKT7. Interaction deficient mutations were generated from corresponding vectors from ref. (6) containing wild-type sequences, using primers harboring desired mutations (***SI Appendix*** **Table S2**) (6). The ΔC construct was made by introducing the same 1-bp deletion as found in the mouse allele, so it fully mimics the truncation and extra amino acids encoded by frame shift. All plasmids were confirmed by sequencing of inserts.

Mating of bait- and prey-containing strains (Y187 and Y2HGold yeast strains) and selection on synthetic dextrose plates lacking tryptophan and leucine and containing 200 ng/ml Aureobasidin A (SD-Trp/Leu/AbA) alone or also lacking adenine and histidine (SD-Trp/Leu/Ade/His/AbA) were performed following the manufacturer's instructions (Clontech).

The presence of the fusion proteins in Y2H strains was verified by immunoblot analysis with antibodies against c-MYC and HA for detecting bait and prey proteins, respectively. To prepare cell-free extracts, yeast cells were harvested, resuspended in 100 µl distilled water, then 100 µl of 0.2 M NaOH was added and the mixture was incubated for 5 min at room temperature. The cell pellets were collected, resuspended in 60 µl SDS sample buffer, and boiled for 5 min. The resulting supernatants were transferred to new tubes and stored at –80 °C or used immediately. 10 µl of supernatant was typically loaded per lane for immunoblot analysis. Samples were separated on 4%–12% Bis-Tris NuPAGE precast gels (Life Technologies) at 130 V for 80 min. Proteins were transferred to polyvinylidene difluoride (PVDF) membranes by wet transfer method in transfer buffer (25 mM Tris, 192 mM glycine, 16% methanol) at 120 V for 70 min. Membranes were blocked with 5% non-fat milk in TBST buffer (20 mM Tris-HCl, pH 7.4, 150 mM NaCl, 0.1% Tween-20) for 40 min at room temperature on an orbital shaker. Blocked membranes were incubated with primary antibodies (mouse monoclonal anti-c-MYC 9E10 (MSK Antibody Core Facility), 1:2000; or mouse monoclonal anti-HA (Covance, MMS-101P), 1:2000) overnight at 4 °C. Membranes were washed with TBST buffer for 40 min at room temperature, then incubated with HRP-conjugated secondary antibody (sheep anti-mouse IgG

(GE Healthcare, NA931V), 1:6,000) for 1 hr at room temperature. Membranes were washed with TBST buffer for 45 min, and the signal was developed by ECL Prime (GE Healthcare) and imaged on a Biorad ChemiDoc MP Imaging System.

Testis immunoprecipitation and immunoblot analyses

Decapsulated testes were snap-frozen in an Eppendorf tube in liquid nitrogen and stored at –80°C until usage. The frozen tissue was disrupted with a plastic pestle in RIPA buffer (50 mM Tris-HCl pH 7.5, 150 mM NaCl, 0.1% SDS, 0.5% sodium deoxycholate, 1% NP40) supplemented with protease inhibitors (Roche Mini tablets). The homogenate was supplemented with Benzonase nuclease (EMD Millipore (70664-3), 28 unit/ml) and 10 mM $MgCl_2$ and incubated with end-over-end rotation for 1 hr at 4 °C. The samples were centrifuged at 21,130 g for 20 min at 4 °C. The supernatant was transferred to a new Lobinding tube (Eppendorf). The extract was pre-cleared with 50 µl protein A Dynabeads (Thermofisher) per sample by end-over-end rotation for 1 hour at 4 °C followed by magnetic removal of the beads. Then, 2 µg of antibodies (guinea pig anti-ANKRD31, rabbit anti-ANKRD31, or rabbit anti-REC114 (6)) were added to pre-cleared lysates and incubated with end-over-end rotation overnight at 4 °C. Protein A Dynabeads (50 µl) were added to the tubes and incubated for 1 hr with end-over-end rotation at 4 °C. Beads were washed three times with 500 µl RIPA buffer, eluted in 1× NuPAGE LDS sample buffer (Invitrogen) with 50 mM DTT, and incubated for 10 min at 70 °C.

Eluted proteins were separated on 3%–8% Tris-Acetate NuPAGE precast gels (Life Technologies) at 150 V for 70 min and were transferred to PVDF membranes by wet transfer method in transfer buffer (192 mM glycine, 25 mM Tris, 10% methanol) at 120 V for 40 min at 4 °C. Membranes were blocked with 5% non-fat milk in 1× phosphate buffered saline (PBS) with 0.1% Tween (PBS-T) for 1 hr at room temperature. Blocked membranes were incubated with primary antibodies (guinea pig anti-ANKRD31, 1:4000; rabbit anti-ANKRD31, 1:4000) overnight at 4 °C. Membranes were washed with PBS-T for 3 × 10 min at room temperature on an orbital shaker, then incubated with HRP-conjugated secondary antibodies (rabbit anti-guinea pig IgG (Abcam 6771), 1:6000; goat anti-rabbit IgG (Biorad 170-6515)) for 1 hr at room temperature. Membranes were washed with PBS-T for 3 × 10 min and were developed by ECL Prime (GE Healthcare) and imaged on a Biorad ChemiDoc MP Imaging System.

Preparation of spermatocyte chromosome spreads

Decapsulated testes were deposited in 50 ml Falcon tubes containing 2 ml TIM (104 mM NaCl, 45 mM KCl, 0.6 mM KH_2PO_4, 1.2 mM $MgSO_4$, 6.0 mM sodium lactate, 1.0 mM sodium pyruvate, 0.1% glucose). Collagenase (200 µl of a 20 mg/ml solution in TIM) was added and incubated at 450 rpm for 55 min at 32 °C in a thermomixer. Samples were filled to a final volume of 15 ml with TIM, then centrifuged for 1 min at 39 g at room temperature. The supernatant was carefully removed with pipettes and the washing was repeated three times. Separated tubules were resuspended in 2 ml TIM, then 20 µl DNase I (400 µg/ml in TIM) and 200 µl trypsin (7 mg/ml in TIM) were added sequentially and incubated on a thermomixer for 15 min at 32 °C at 450 rpm. Trypsin inhibitor (500 µl of a 20 mg/ml solution in TIM) or FBS and 50 µl of the DNase I solution was added to terminate the reaction. A transfer pipet was used to further disperse the tissue by pipetting up and down for about 2 min. Cell suspensions were then filtered through a 70-µm cell strainer into a new 15 ml Falcon tube. TIM was added to a final volume of 15 ml and was centrifuged for 5 min at 108 g. The cell pellet was resuspended with 15 µl DNase I solution and TIM was added to a final volume of 15 ml. The washing procedure was repeated 2 times. The final pellet was resuspended in TIM according to the original testis weight (~100 mg in 10 ml). This single-cell suspension (500 µl) was transferred to an Eppendorf tube

and centrifuged for 3 min at 845 g. The cell pellet was resuspended in 40 µl of freshly prepared 0.1 M sucrose and incubated for 8 min at room temperature. Edges of superfrost glass slides were covered by Immedge pen, and each slide received 85 µl of 1% paraformaldehyde (PFA) (dissolved in presence of NaOH at 65 °C, 0.15% Triton, pH 9.3, filtered through a 0.22 µm filter, kept at –20 °C until usage). After incubation, 20 µl of cell suspension in the sucrose solution was added, slides were swirled three times, and dried in a closed slide box for 2 hr, followed by drying with a half-open lid for 1 hr at room temperature. Slides were washed in a Coplin jar on a shaker 1 × 5 min in milli-Q water and 3 × 5 min with 0.4% PhotoFlow (Kodak), then air-dried. Slides were stained immediately or were wrapped in aluminum foil and stored at −80°C.

Histology

Testes and epididymides dissected from adult mice were fixed in Bouin's fixative for 4 to 5 hr at room temperature, or in 4% PFA overnight at 4 °C. Bouin's fixed testes were washed in 15 ml milliQ H_2O on a horizontal shaker for 1 hr at room temperature, followed by five 1-hr washes in 15 ml of 70% ethanol on a roller at 4 °C. PFA-fixed tissues were washed 4 × 5 minutes in 15 ml milliQ H_2O at room temperature. Fixed tissues were stored in 70% ethanol before embedding in paraffin and sectioning (5 µm for testes, 8 µm for ovaries). The tissue sections were deparaffinized with EZPrep buffer (Ventana Medical Systems). Hematoxylin and eosin (H&E) staining, periodic acid Schiff (PAS) staining and immunohistochemical TUNEL assay were performed by the MSK Molecular Cytology Core Facility using the Autostainer XL (Leica Microsystems, Wetzlar, Germany) automated stainer for H&E with hematoxylin counterstain, and using the Discovery XT processor (Ventana Medical Systems, Oro Valley, Arizona) for TUNEL. The detection was performed with DAB detection kit (Ventana Medical Systems) according to manufacturer's instructions. Slides were counterstained with hematoxylin and coverslips were mounted with Permount (Fisher Scientific).

Whole H&E or TUNEL stained slides were scanned and digitized with the Panoramic Flash Slide Scanner (3DHistech, Budapest, Hungary) with a 20× 0.8 NA objective (Carl Zeiss, Jena, Germany). High-resolution images of H&E and IHC were acquired with a Zeiss Axio Imager microscope using a 63× 1.4 NA oil immersion objective (Carl Zeiss, Jena, Germany).

Exo7/T-seq

Agarose plugs containing genomic DNA were prepared as previously described (32). To do this, testes from 14.5-dpp juvenile mice were decapsulated and incubated in DMEM containing 0.1% polyvinyl alcohol (PVA, Sigma), 0.1% BSA (Gibco) with 1 mg/ml collagenase type IV (Worthington) and 1 mg/ml Dispase II (Sigma) for 20 min at 35 °C in a thermomixer at 450 rpm. Seminiferous tubules were then rinsed three times and further treated with trypsin (TrypLE™ express enzyme, Gibco) and 1 µg/ml DNase I (Roche) for 15 min at 35 °C in a thermomixer at 450 rpm. Trypsin was inactivated with 5% FBS and tubules were further dissociated by gentle pipetting. Cells were passed through a 70-µm cell strainer (BD Falcon) and washed three times in GBSS containing 0.1% PVA.

Cells were embedded in plugs of 1% low-melting-point agarose (Lonza) in GBSS (1.5 million to 2 million cells per plug). Plugs were incubated with 100 µg/ml proteinase K (Roche) in lysis buffer (0.5 M EDTA at pH 8.0, 1% N-lauroylsarcosine sodium salt) at 50 °C over two nights. Plugs were washed 5 × 20 min with TE (10 mM Tris-HCl at pH 7.5, 1 mM EDTA at pH 8.0), and then incubated with 100 µg/ml RNase A (Thermo) for 3 hr at 37 °C. Plugs were then washed five times with TE and stored in TE at 4 °C until usage.

To prepare sequencing libraries, plugs were washed in 1 ml of Exo VII buffer (50 mM Tris-HCl, 50 mM sodium phosphate, 8 mM EDTA, 10 mM 2-mercaptoethanol, pH 8.0) 2 × 15

min, equilibrated with 50 U of exonuclease VII (NEB) in 100 µl of Exo VII buffer for 10 min on ice and then incubated for 60 min at 37 °C using a thermomixer at 400 rpm. Plugs were rinsed with TE and then washed with 1 ml NEB buffer 4 (NEB) 3 × 15min, equilibrated with 75 U of exonuclease T (NEB) on ice for 30 min, and then incubated for 90 min at 24 °C using a thermomixer at 400 rpm. Plugs were washed in 500 µl of 1× T4 polymerase buffer (1× T4 ligase buffer (NEB) supplemented with 100 µg/ml BSA and 100 µM dNTPs (Roche)) 4 × 30 min on ice. The wash solution was then replaced with 1× T4 polymerase buffer containing 30 U T4 DNA polymerase (NEB) and incubated at 12 °C for 30 min. P5 adapters were ligated to the ends with 1 µl of 2000 U/µl T4 DNA Ligase (NEB) at 16 °C for 20 hr. After ligation, plugs were washed in 1 ml TE three times and incubated in TE overnight at 4 °C.

Plugs were washed with 500 µl of 1× β-agarase I buffer (10 mM Bis-Tris-HCl, 1 mM EDTA, pH 6.5) on ice for 30 min, then 150 µl of 1× β-agarase I buffer was added and incubated at 70 °C for 5 min to melt the agarose, followed by vortexing for 30 s and brief centrifugation. The vortex and spin-down procedure was repeated three times. Samples were cooled to 42 °C and 2 µl of β-agarase I enzyme (NEB) was added and incubated at 42 °C for 90 min with occasional vortexing. DNA was sheared into fragment sizes ranging 200–500 bp with a Covaris system (E220 Focused-ultrasonicator, microtube-500) using the following parameters: delay 300 s then three cycles of [peak power 175, duty factor 20, cycles/burst 200, duration 30 s, and delay 90 s]. Sonicated samples were added to 1250 µl of 100% ethanol and 55 µl of 3 M sodium acetate and incubated overnight at –20°C.

Ethanol precipitated DNA was dissolved in 52 µl of TE for 1 hr at 37 °C. SPRIselect beads (Beckman Coulter) were used to remove unligated adapters. Fragments containing the biotinylated adapter were purified with Dynabeads™ M-280 streptavidin (Thermo Fisher). The end-repair reaction was done using the End-it DNA end-repair kit (Lucigen). P7 adapters were ligated to DNA fragments as described above. PCR was done on beads. PCR products were purified with 0.9× AMPure XP beads (Beckman Coulter) to remove primer dimers and unligated adapters. DNA was sequenced on the Illumina HiSeq platform in the Integrated Genomics Operation at MSK. We obtained paired-end reads of 50 bp.

Bioinformatic analysis was performed as described previously (31, 32). In brief, Trim Galore <http://www.bioinformatics.babraham.ac.uk/projects/trim_galore/> was used to trim and filter reads with the arguments -paired -length 15. Bowtie2 (40) was run with the arguments -N 1 -X 1000 to map sequence reads onto the mouse reference genome (mm10). Picard <https://broadinstitute.github.io/picard/> eliminated reads that were duplicated. With the -q 20 argument, Samtools (42) extracted properly mapped and uniquely mapped reads (MAPQ ≥ 20). Reads for Exo7/T-seq were counted at the nucleotide immediately adjacent to the position where the biotinylated adapter DNA was mapped (i.e., corresponding to the last position of the ssDNA). Maps were analyzed using R (versions 3.3.1 and 4.0.3).

Uniquely mapping fragments derived from Exo7/T-seq were used to identify hotspot locations (peak calling). Peak calling was performed using MACS (v.2.2.7.1) (43) with the following parameters: -g mm -- keep-dup all -- nolambda -- nomodel. Numbers of peak calls are indicated in ***SI Appendix*** **Table S1**. Narrow peaks were then widened by 5 kb and merged. Then merged peaks were compared with PRDM9-targeted hotspots (n=13,960 from SPO11 oligo sequencing) or the 10,000 hottest of previously defined default hotspots (12, 34) to calculate the fraction of hotspot usage. Overlaps were defined as peaks with a shared region of at least 1 bp.

Supplemental References

41. P. J. Romanienko et al., A vector with a single promoter for *in vitro* transcription and mammalian cell expression of CRISPR gRNAs. *PLoS One* **11**, e0148362 (2016).

42. H. Li et al., The Sequence Alignment/Map format and SAMtools. *Bioinformatics* **25**, 2078–2079 (2009).

43. Y. Zhang et al., Model-based analysis of ChIP-Seq (MACS). *Genome Biol*. **9**, R137 (2008).

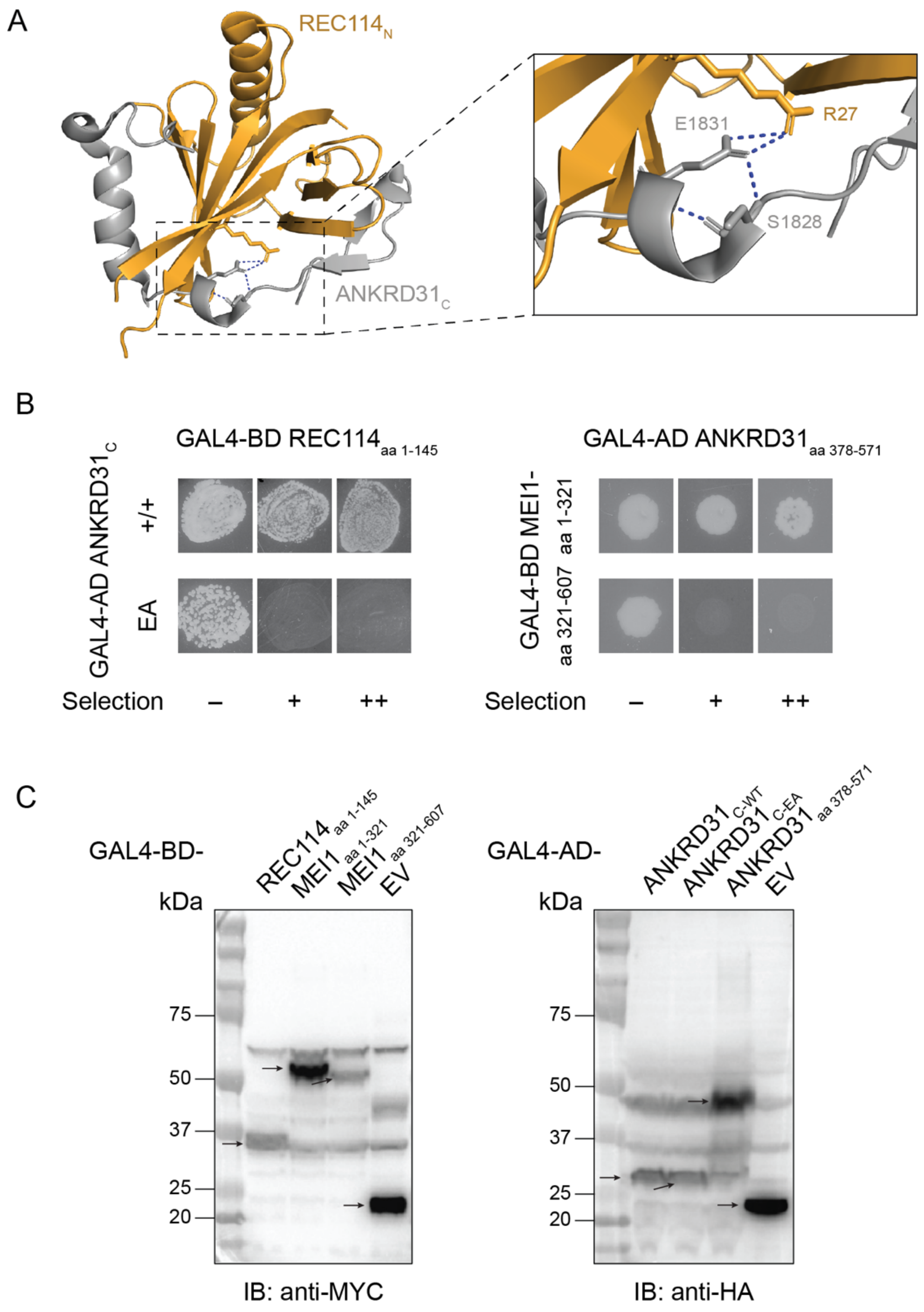

Figure S1. ANKRD31 interactions with REC114 and MEI1. (A) Crystal structure of ANKRD31$_C$ (gray) bound to the REC114$_N$ pleckstrin homology domain (yellow) (PDB: 6NXF). The zoomed detail highlights contacts made by ANKRD31 Glu-1831. (B) Y2H interactions of REC114 aa 1-145 and wild type or EA mutant ANKRD31$_C$ and Y2H interactions of full-length

and fragments of ANKRD31 and MEI1. Cells express the indicated Gal4 activating domain (AD) and binding domain (BD) fusions. EV, empty vector. “Selection” indicates amino acid dropouts and aureobasidin to detect reporter activation at moderate (+) and high (++) stringency. (C) Immunoblots probed with anti-MYC and anti-HA antibody to detect the expression of GAL4-BD and GAL4-AD fusion proteins, respectively, in (B). The arrows indicate expected band positions. EV, empty vector.

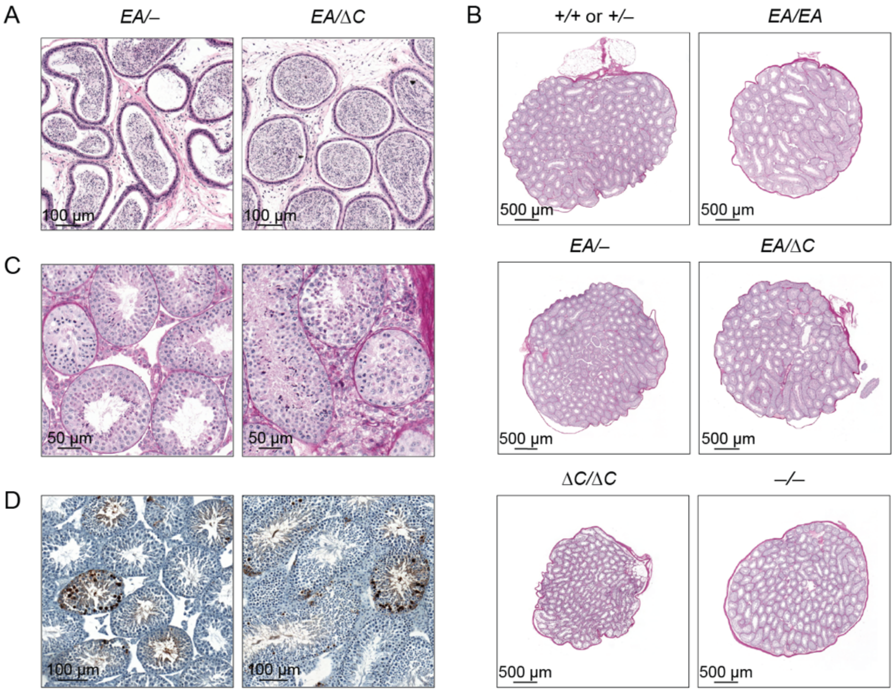

Figure S2. Hypogonadism and infertility in an interaction-deficient *Ankrd31* allelic series. (A) Sections of epididymides from adult mice (2–8 mos old), PFA fixed and H&E stained. (B-C) Sections of adult (2–8 mos old) testes (B) and seminiferous tubules (C), Bouin's fixed and PAS stained. (D) Adult (2–8 mos old) testis sections were stained with TUNEL and hematoxylin.

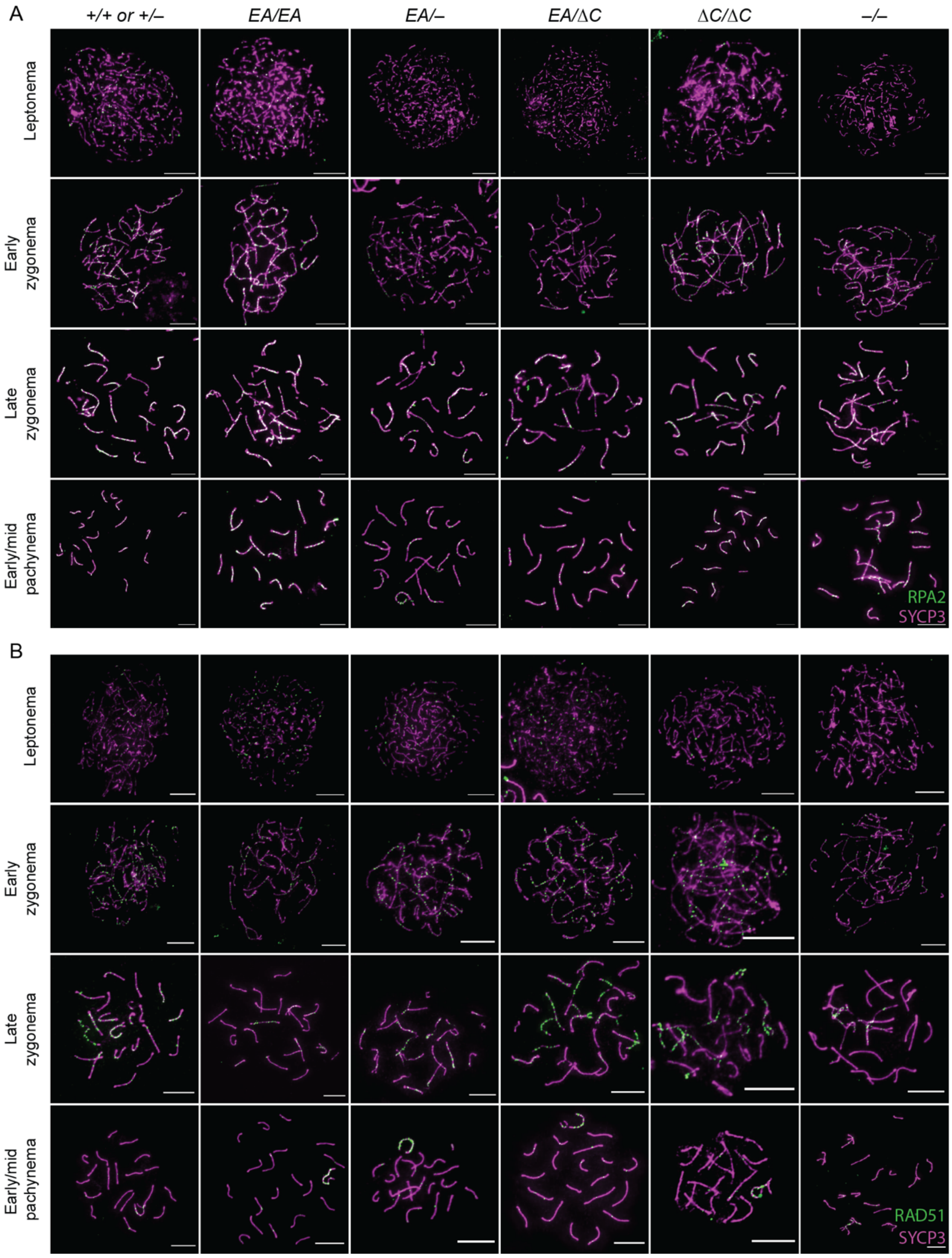

Figure S3. ANKRD31–REC114 interaction deficiencies cause progressive defects in DSB formation and recombination. Representative images of RPA2 staining (A) or RAD51 staining (B) of spermatocyte chromosome spreads. Scale bars, 10 μm.

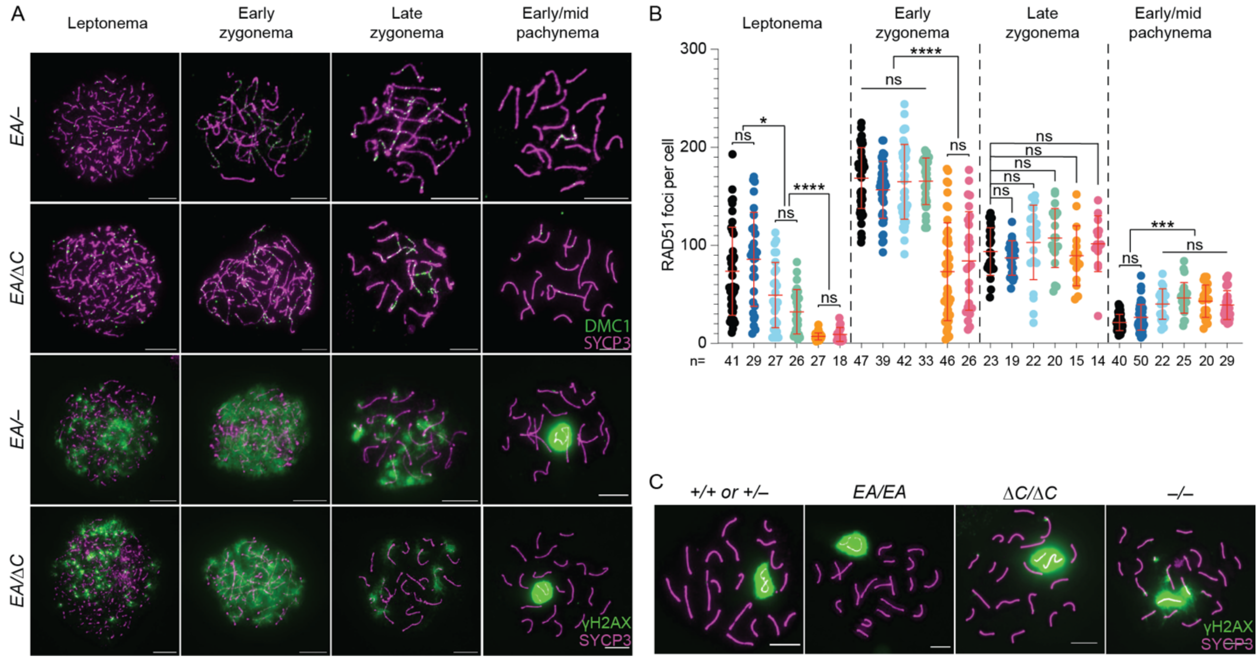

Figure S4. ANKRD31–REC114 interaction deficiencies cause progressive defects in DSB formation and recombination. (A) Representative DMC1 (top two rows) and γH2AX staining (bottom two rows) of *Ankrd31*$^{EA/–}$ and *Ankrd31*$^{EA/\Delta C}$ spermatocyte chromosome spreads. (B) Quantification of focus numbers of RAD51. Each point is the count from one cell (total cell numbers are given below the graphs) from three or more animals of each genotype. The red lines are means. The results of two-tailed Mann-Whitney U tests are shown: ns, not significant ($p > 0.05$), $*p \leq 0.05$, $**p \leq 0.01$, $***p \leq 0.001$, and $****p \leq 0.0001$. (C) Representative γH2AX staining of pachytene spermatocyte chromosome spreads. Scale bars in A and C, 10 μm.

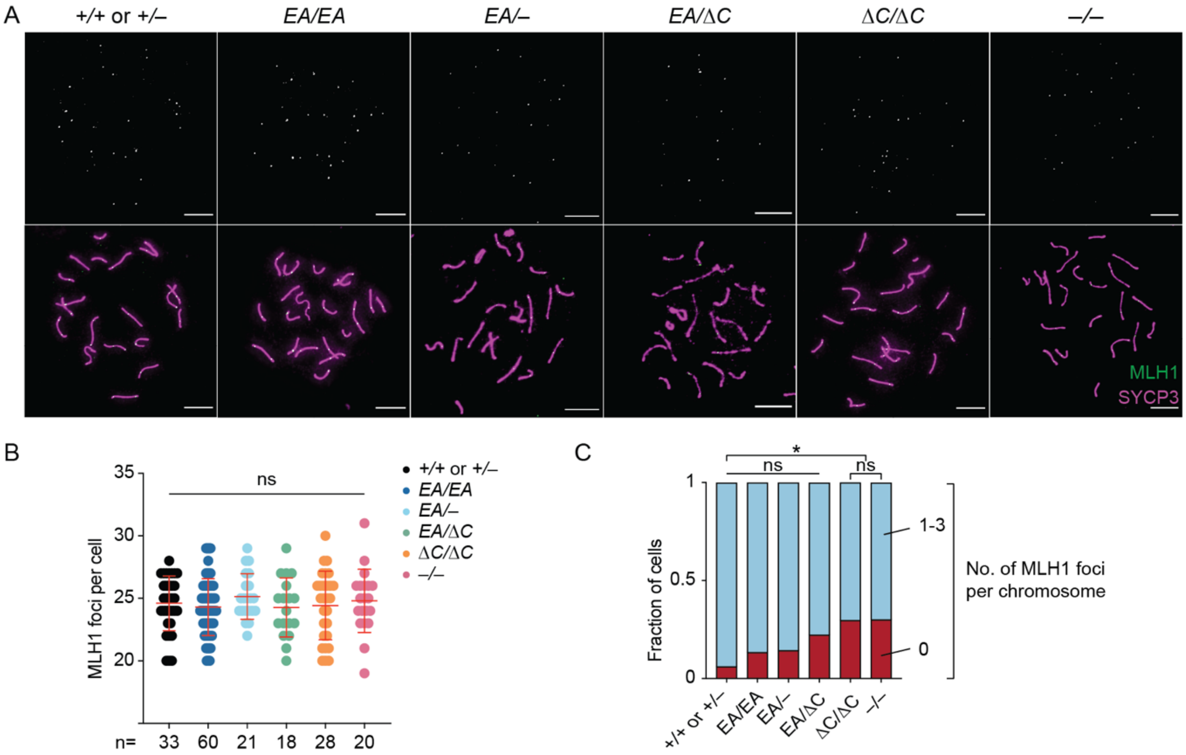

Figure S5. Autosomal recombination. (A) Representative pachytene cells labeled with anti-SYCP3 and anti-MLH1 antibodies. Top row, MLH1 staining only; bottom row, merge of MLH1 and SYCP3 channels. Scale bars, 10 µm. (B) Quantification of MLH1 foci from two or more animals of each genotype. The number of cells analyzed is indicated below. The red lines are means ± SD. The results of two-tailed Mann-Whitney U tests are shown: ns, not significant ($p > 0.05$). (C) Quantification of percent of cells containing autosomes with the indicated number of MLH1 foci in pachynema (same cells as presented in panel B).

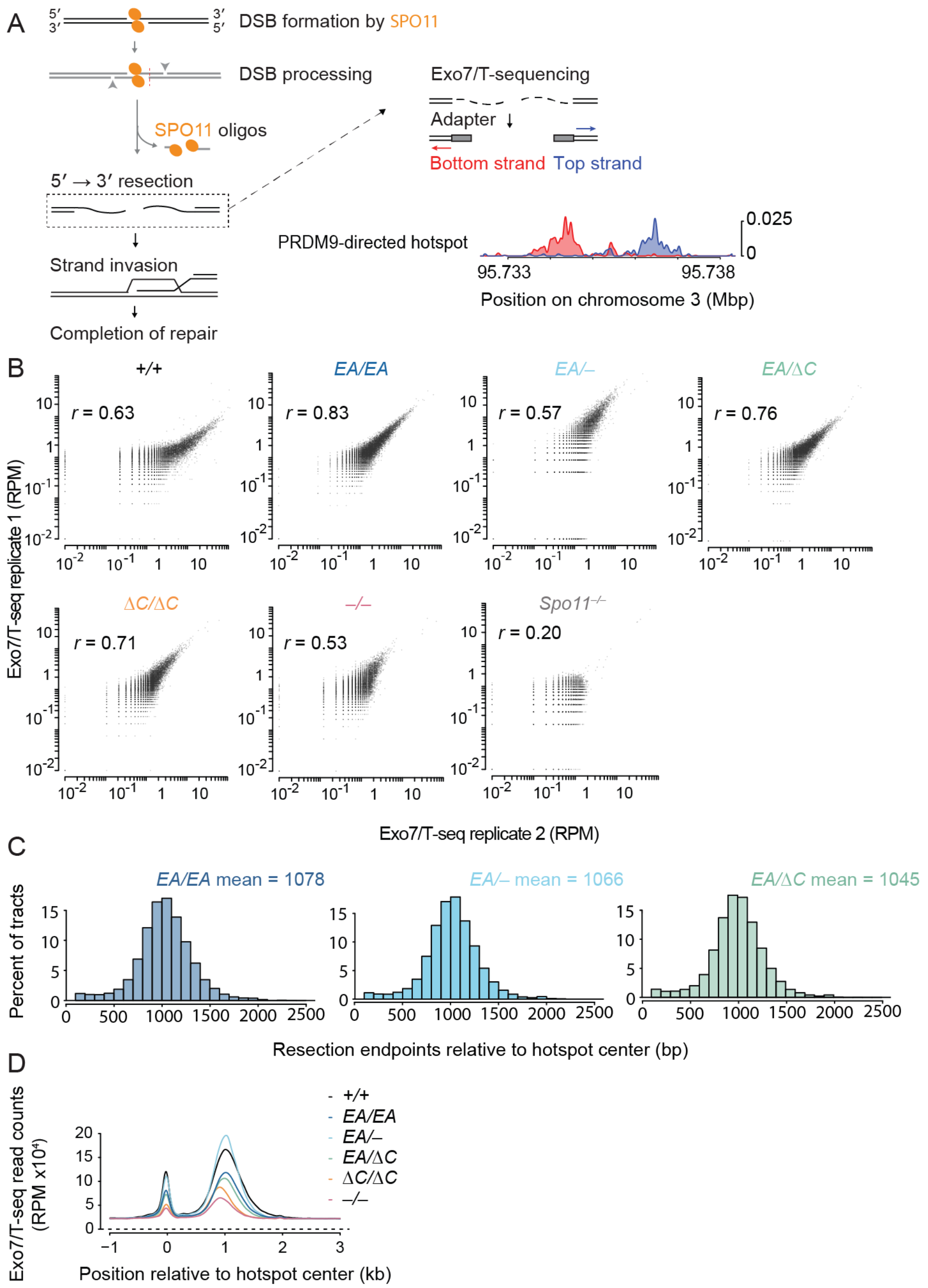

Figure S6. Schematic of Exo7/T sequencing and resection length. (A) Steps in DSB formation and processing and schematic of Exo7/T sequencing to detect resection endpoints. An example profile is shown from wild type at a PRDM9-directed hotspot in B6 mice. (B) Correlation (Pearson's *r*) of Exo7/T-seq read counts between biological replicates. Each point is a SPO11-oligo hotspot, with Exo7/T-seq signal summed from −2000 to −250 bp (bottom strand) and +250 to +2000 bp (top strand) around hotspot centers. Hotspots with no signal (or 0 RPM) were excluded for Pearson's *r* calculation. Hotspots with $\leq 10^{-2}$ RPM were set as 10^{-2} for plotting purposes only. Note that the correlation for the *Spo11*$^{-/-}$ replicates is poor because the sequencing signal represents mostly dispersed, nonspecific background. (C) Resection length distributions in *Ankrd31*$^{EA/EA}$, *Ankrd31*$^{EA/-}$ and *Ankrd31*$^{EA/\Delta C}$. (D) Non-normalized, averaged

Exo7/T-seq signals around PRDM9-directed hotspots (n = 13,960 SPO11-oligo hotspots in the C57BL/6J strain (34)). The bottom-strand reads were flipped and combined with the top-strand reads.

Table S1. Overlap of Exo7/T-seq peak calls with SPO11-directed or default hotspots.

Overlap of Exo7/T-seq peak calls with SPO11-directed or default hotspots

	Total peak called	Merged peaks	Overlap with SPO11-directed hotspots	Overlap with default hotspots	Overlap with both	Overlap with neither
Ankrd31$^{+/+}$	5637	2704	2443	86	77	252
Ankrd31$^{EA/EA}$	6621	3213	3046	98	96	165
Ankrd31$^{EA/–}$	3743	2075	1975	59	57	98
Ankrd31$^{EA/\Delta C}$	5551	2931	2583	111	80	317
Ankrd31$^{\Delta C/\Delta C}$	6108	3562	1700	1432	99	529
Ankrd31$^{–/–}$	2899	1872	1187	400	41	326
Spo11$^{–/–}$	211	97	15	2	0	80

Table S2. Primers for genotyping and cloning.

**Primers for genotyping**		
Genotype	Primer Name	Primer Sequence
Ankrd31^{EA}	18MGAnkF08	5'-CCGATCACGTATGCTCTGATACGG -3'
	18MGAnkR05	5'-ACTGACCATACGAGTTATAGTGCAGG -3'
Ankrd31$^{\Delta C}$	18MGAnkF05	5'-AGCAGGTACTTCCAGAGAGTCGAT -3'
	18MGAnkR03	5'-GCTAACTAAGCTACCAAGAAAGCAGAGC -3'
**pGAD-ANKRD31 cloning primers**		
Ankrd31^{EA}		5'-CTTCAAATCAGCAAGCAGGCGC-3'
		5'-CATTACATGGCATGGCAGAAGCGCC-3'
Ankrd31$^{\Delta C}$		5'-CAGAAGCAGGAGCTTCTGCCATGCCATGTAATGGAGCAG-3'
		5'-AGAAGCTCCTGCTTCTGATTTGAAGTATCTCTTTTATCTGT-3'

Table S3. Antibodies.

**Primary Antibodies**					
Target	Dilution for IF	Dilution for immunoblot	Host species	Supplier	Catalog number
γH2AX	1:6000	n/a	Rabbit	Abcam	ab2893
DMC1 (H100)	1:100	n/a	Rabbit	Santa Cruz	sc-22768
MLH1	1:25	n/a	Mouse	BD-Pharmingen	51-1327GR
RPA2	1:1000	n/a	Rabbit	Abcam	ab76420
RAD51	1:100	n/a	Rabbit	Aviva	ARP33450
SYCP3 (D-1)	1:300	n/a	Mouse	Santa Cruz	sc-74569
SYCP3	1:200	n/a	Rabbit	Nova	NB300-232
SYCP1	1:200	n/a	Rabbit	Abcam	ab15090
REC114	1:200	n/a	Rabbit	Stanzione et al., 2016	n/a
ANKRD31	1:200	1:4000	Guinea Pig	Boekhout et al., 2019	n/a

**Secondary Antibodies**					
Target	Dilution for IF	Fluorophore	Host species	Supplier	Catalog number
anti Guinea pig	1:400	Alexa-488	Goat	Mol Probes	A-11073
anti Mouse	1:400	Alexa-594	Goat	Mol Probes	A-11005
anti Mouse	1:400	Alexa-594	Donkey	Life Technologies	A-21203
anti Mouse	1:400	Alexa-488	Donkey	Life Technologies	A-21202
anti Rabbit	1:400	Alexa-594	Goat	Invitrogen	A-11012
anti Rabbit	1:400	Alexa-488	Goat	Invitrogen	A11034
anti Rabbit	1:400	Alexa-488	Donkey	Thermofisher Scientific	R37118
